# Plants Used for Treating Hypertension Among Ethnic Groups in Northern Thailand

**DOI:** 10.3390/plants14071066

**Published:** 2025-03-30

**Authors:** Prattana Sumridpiem, Henrik Balslev, Pimonrat Tiensawat, Oratai Neamsuvan, Angkhana Inta

**Affiliations:** 1 Department of Biology, Faculty of Science, Chiang Mai University, Chiang Mai 50200, Thailand; prattanacmu@gmail.com (P.S.); pimonrat.t@cmu.ac.th (P.T.); 2 Department of Biology, Aarhus University, Building 1540, Ny Munkegade 116, DK-8000 Aarhus C, Denmark; henrik.balslev@bio.au.dk; 3 Forest Restoration Research Unit (FORRU), Faculty of Science, Chiang Mai University, Chiang Mai 50200, Thailand; 4 Environmental Science Research Center, Faculty of Science, Chiang Mai University, Chiang Mai 50200, Thailand; 5 Faculty of Traditional Thai Medicine, Prince of Songkla University, Songkhla 90110, Thailand; oratai.n@psu.ac.th

**Keywords:** anti-hypertensive, ethnobotanical knowledge, high blood pressure, medicinal plants

## Abstract

The incidence of hypertension (HT) is rapidly increasing globally, and it is considered to be a critical public health problem. Due to the demand for medication and because various side effects of anti-hypertensive drugs have been reported, complementary and alternative therapies, including Thai Indigenous medicine (TIM), should be explored for treating HT. Medicinal plants traditionally used by multiple cultures over long time periods in HT treatment are more likely to be pharmacologically active and might provide useful data, leading to anti-hypertensive drug discovery. Ethnomedicinal field observations were undertaken with 41 key informants in eleven villages in Chiang Mai province from December 2022 to November 2023. In addition, we gathered data on traditional plants used for treating HT among 12 ethnic groups from 41 original references published between 1987 and 2023, covering nine provinces in northern Thailand. Important species among plants used for treating hypertension were identified by calculating their relative frequency of citation (RFC). In total, we found 237 plant species that were used for treating HT. Of these, 173 species had already been reported in the literature, and 96 species were documented in our fieldwork. There were 30 plant species from our field survey that overlapped with species accounted for in the literature. Fabaceae was the plant family with the most species (23 sp, 10%) used for treating HT. The most commonly used species was *Blumea balsamifera* (L.) DC. (Asteraceae), and it had the highest recorded RFC value of 0.1979. There were 64 plant species that were reported for the first time for HT treatment among ethnic groups in northern Thailand. Of the recorded species, 24 were particularly promising in the treatment of HT, and their efficacy was confirmed by comparing our results to previous studies of plants with anti-hypertensive properties.

## 1. Introduction

Hypertension (HT) is a serious global public health problem that needs research attention.

A report from Thailand’s Ministry of Public Health showed that HT is the main cause of more than 50,000 deaths annually in Thailand [1,2]. Hypertension is caused by unhealthy lifestyles (e.g., being overweight, too much salt in the diet, lack of exercise, alcohol, caffeine, and smoking), internal factors (e.g., stress), concomitant diseases (e.g., heart failure, coronary heart disease, kidney failure, and stroke), and others (e.g., age and gender) [1,2,3]. It leads to death and disability worldwide. In modern Thai or Western medicinal practice, blood pressure is a physical value that can be measured with a scientific tool called a sphygmomanometer. A patient who has HT or high blood pressure has a corresponding measured systolic blood pressure (SBP) ≥ 140 mmHg and/or diastolic blood pressure (DBP) ≥ 90 mmHg [1]. Hypertension is broadly classified into two types based on its underlying causes. Primary hypertension or essential hypertension (90–95% of cases) usually has no specific origin. Secondary hypertension (5–10% of cases) is caused by other physiological disorders, particularly in the kidneys, arteries, heart, or endocrine system [1,2,4]. The symptoms of HT are headache, dizziness, blurred vision, palpitation, tinnitus, and nose bleeding [5]. As long as the various adverse effects of anti-hypertensive drugs are prominent, including limitations on cost [3], complementary and alternative therapies, including Thai Indigenous medicine (TIM), should be explored for treating HT.

In pharmacology, the drugs that are used to reduce blood pressure belong to five types, including diuretics, angiotensin-converting enzyme inhibitors (ACEIs), angiotensin receptor blockers (ARBs), beta blockers, and calcium channel blockers (CCBs). These drugs affect the organs differently, and together, they are classified as anti-hypertensive drugs. Modern medicine aspires to reduce blood pressure as the final result of HT treatment [1]. However, a persistent cough is a common side effect of ACEIs. The incidence of cough varies from 23.6 to 31.3% [3].

Cardiovascular diseases, including HT and paralysis after stroke, were ranked fifth (62.5%) among the diseases that were treated within the Thai traditional medicine system [3]. However, evidence of the conceptual knowledge of HT has never been recorded in Thai Indigenous medicine (TIM) [6]. Traditional healers in southern Thailand assume that high blood pressure is derived from internal factors, such as the abnormality of wind and fire elements in the body, and external factors, such as food, weather, etc. [7,8].

Northern Thailand is well known for its traditional knowledge, cultural diversity, plant biodiversity, ecological diversity, and numerous ethnomedicinal studies [9]. There are several ethnobotanical works that focus on medicinal plants, mostly investigating their use for systemic disease groups such as women’s health [10,11], digestive disorders [12], infectious diseases [13], and musculoskeletal disorders [14]. However, plants for treating HT among Indigenous people are only rarely mentioned at the local level. In Thailand, the highest ethnic diversity is found in the northern provinces. Moreover, Chiang Mai province in northern Thailand is home to most of the ethnic minorities who live in the highlands [15,16]. In this study, we collected data on medicinal plants used by 12 different ethnic groups in northern Thailand. The Karen, Lisu, Palaung, and Tai Yai came from Myanmar, and the Hmong, Mien, Akha, Lahu, Haw, and Lisu migrated from China centuries ago [16,17]. The origin of the Lawa or Lua has a long history, which is poorly understood. They may have migrated from southern Thailand or Cambodia to northern Thailand some 2000 years ago [18,19]. The Tai Yuan or Thai Lanna are also known as Khon Mueang, and they are the majority ethnic group, and they inhabit a large region of northern Thailand [5]. The Indigenous medicine of ethnic communities in northern Thailand is a prime representation of traditional knowledge, cultural diversity, high biodiversity, and ecological diversity [9]. Traditional Indigenous medicine (TIM) is defined as the examination, diagnosis, therapy, treatment, prevention of disease, promotion, and rehabilitation of health using the traditional inherited knowledge within local communities [2]. It is often assumed that the high degree of agreement in uses could be related to the presence of bioactive compounds [13].

Therefore, our research aimed to investigate the ethnomedicinal plant species used for treating HT among ethnic communities in northern Thailand and evaluate the important medicinal plants with high potential for HT treatment. Specifically, we asked the following questions: (1) Which plants are used to treat HT by the ethnic communities in northern Thailand?; (2) Which plant parts are used and how are they prepared and applied?; (3) Which type of medicine is the most important for the treatment of hypertension?; (4) Which plant species are the most important for the treatment of hypertension?; and (5) Should these important species be recommended for hypertension treatments based on scientific evidence?

## 2. Results

### 2.1. Medicinal Plants Used for Treating Hypertension in Northern Thailand

**Figure 1:**
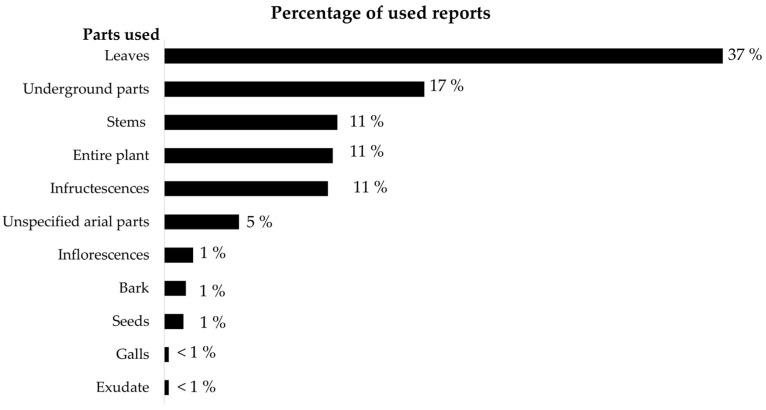
Percentage of use reports for each plant part of medicinal plant species used for treating HT among 12 ethnic groups in northern Thailand.

Our combined dataset, including both literature references and new records obtained through fieldwork, covers 237 species in 194 genera and 82 plant families, which, together, have 659 use reports (Table 1, Figure 1). Most of them are flowering plants, but there are also two species of gymnosperms (Pinaceae), three ferns (Marsileaceae, Ophioglosaceae, and Polypodiaceae), and one lichen (Usneaceae). The life forms include 85 species of herbs, 60 species of shrubs, 39 species of climbers, 42 species of trees, and 7 species of grasses. Among the families, Fabaceae (23 species), Asteraceae (17 species), Acanthaceae (14 species), Lamiaceae (12 species), and Zingiberaceae (11 species) are those with the most species used in the treatments of HT (Table 2). Of the 82 different plant families, 42 are represented by a single species (Table S1). Among the 12 ethnic groups for which we had literature-based and field-based data, the Tai Yuan use the highest number of plant species (121) and have the most use reports (206) for treating HT (Table 1).

Our fieldwork reveals 64 species that had not been previously reported for HT treatment among ethnic groups in northern Thailand (Table S1). These new records comprise 27% of the total list of species used to treat HT, including both previous records (Table S2) and new field records gathered for this study. Of all 64 new records of medicinal plant species used to treat HT in northern Thailand, the Tai Yai knew 26 species (with 53 use reports), the Hmong knew 23 species (with 65 use reports), the Lisu knew 12 species (with 42 use reports), and the Karen knew 10 species (with 17 use reports).

### 2.2. Plant Parts Used and Their Application

Leaves are the most commonly used part of plants for treating HT. They are mentioned in 37% of all use reports, followed by underground parts (17%) and stems (11%) (Figure 1, Supplementary Table S1). There are many methods for preparing medicinal plants (Table 3, Supplementary Table S1). Some of the use reports mentioned more than one method of preparation and application. Decoction and fresh plants are the most commonly used, contributing 49% and 11%, respectively, of the total use reports. There are different ways of using the plants. Oral ingestion is the most common method (55%), followed by herbal steaming (9%) and bathing (7%). Wiping the body, blowing smoke into ears, and fumigating (burning dried materials and fumigating with the smoke) are all used but much more rarely (Table 4).

### 2.3. Forms of Plant Used

Our data on HT treatment by plants among twelve ethnic groups show that there are three basic ways in which plants are used (as single herbs, herbal recipes, and food) (Table 5). The single herb is a form that uses only one species. Herbal recipes are a form that has more than two species as ingredients. Food is a form that uses plants by cooking them and eating them as health food. Herbal recipes are the most favored form of plant use (Table S1), and it includes 130 plant species and covers 292 use reports. The Tai Yai recommend 43 plant species (107 use reports), and the Tai Yuan recommend 88 plant species (88 use reports) to be used in herbal recipe forms. *Blumea balsamifera* (L.) DC. is the most commonly mentioned plant in herbal recipes, and it is mentioned in 17 use reports. There are 123 plant species (305 use reports) that are used in the single-herb treatments. The Tai Yuan recommend 61 plant species (109 use reports) for single-herb forms. *Morinda citrifolia* L., *Moringa oleifera* Lam., and *Phyllanthus amarus* Schumach. & Thonn. are the most favored plants in the 109 use reports. Lastly, the plants cooked as health foods include 29 species (56 use reports), including *Artemisia lactiflora* Wall. ex DC. (7 use reports), *Basella alba* L. (4 use reports), etc. The Hmong recommend 15 plant species (30 use reports) for the health food form.

### 2.4. Important Species for the Treatment of Hypertension

The values of the frequency of citation (FC) range from 1 to 19, and the degree of relative frequency of citation (RFC) range from 0.0104 to 0.1979. The top 10 medicinal plants in terms of the FC value (Table S1) are *Blumea balsamifera* (L.) DC., followed by *Zingiber purpureum* Roscoe, *Mimosa pudica* L., *Centella asiatica* (L.) Urb., *Thunbergia laurifolia* L., *Andrographis paniculata* (Burm.f.) Nees, *Pinus latteri* Mason, *Phyllanthus amarus* Schumach. & Thonn., *Biancaea sappan* (L.) Tod., *Morus alba* L., *Pinus kesiya* Royle ex Gordon, *Houttuynia cordata* Thunb., *Cymbopogon citratus* (DC.) Stapf, and *Saccharum officinarum* L. However, 143 (65%) out of the total 237 plant species concerning HT treatment were recorded with a degree of frequency or an FC = 1 (Table S1).

## 3. Discussion

### 3.1. Medicinal Plants Used to Treat Hypertension in Northern Thailand 

There is a high diversity of medicinal plants used for treating HT among the 12 ethnic communities in northern Thailand. In total, 38 of the 64 plant species have an FC = 1, and they were reported by only one healer. This shows that these healers have their own particular knowledge concerning the treatment of HT, and their knowledge is not similar to that of any of the other healers. This suggests that the knowledge of plants used could originate independently in individual villages [20] since each ethnic group has different knowledge about the usefulness of plants. This may have resulted from cultural differences or the consistency of the knowledge and experience of the individual traditional healers [7,14,21].

Healers’ knowledge in northern Thailand has been passed down from generation to generation. The healers use plants after observing visible clinical symptoms. While collecting data in the field, we learned that healers provide treatment for patients after observing and listening to their symptoms, considering any additional history, undertaking a traditional physical examination, and making a traditional ethnic medicinal diagnosis. After that, the healers select the specific herb for HT patients based on their Indigenous medical knowledge. The healers use different herbs in their treatment, depending on the knowledge inherited from their ancestors and their own experience.

The employment of 38 different plant species suggests that there are differences in the empirical knowledge held by different healers in southern and northern Thailand, possibly because the ecology of northern and southern Thailand is different and heavily influences the variety of local plants. When comparing the plant species (Supplementary Table S1) used in northern and southern regions, we found that there is a 99% difference. The 1% shared species is *Plumbago indica* L. The ecological difference means that local healers in the north and the south received different empirical knowledge about the plants used in HT treatment. However, this difference is not related to the visible clinical symptomatology. The clinical symptoms of HT patients are mostly similar, even if most of them do not necessarily have the same symptoms. Hypertension (HT) patients sometimes do not have all the symptoms of the condition. They may only present some of the symptoms when they go to see a healer.

Meanwhile, there are 93 plant species used by 12 ethnic groups in northern Thailand with an FC > 1. These are the primary plants that we recommend for further research because plants with a high degree of agreement in uses could possess bioactive compounds [13]. Our data point to the difference in medical knowledge between northern and southern populations. For example, in the case of *Blumea balsamifera* (L.) DC. (FC = 19), the northern and southern healers have different traditional medical knowledge. The folk healers in Songkhla use the fresh or boiled leaves of *Blumea balsamifera* (L.) DC. to stimulate the circulatory system in the treatment of hypertension. In northern Thailand, the Tai Yai healers boil the leaves of *Blumea balsamifera* (L.) DC. for oral ingestion, herbal steaming, and bathing (body, head, and face). The Karen healers boil the leaves of *Blumea balsamifera* (L.) DC. for steaming and burn the leaves for poultices. The Hmong healers pound the leaves of *Blumea balsamifera* (L.) DC. for steaming and burn the leaves for poultices. The Lisu healers soak the fresh leaves of *Blumea balsamifera* (L.) DC. with hot water to wipe the body. When using traditional medicinal knowledge, northern healers perform various techniques for medicinal plant preparation more than southern healers.

Fabaceae are the family with the most species (Table 2) used for treating HT among the 12 ethnic communities in northern Thailand. Fabaceae dominate ethnomedicinal studies in Thailand [14,15,22]. Also, it is one of the largest plant families globally [23]. In Thailand, this is an important family, which is much used in traditional medicine [9,15]. The bioactive compounds in this plant family could include the alkaloid L-mimosine. This bioactive compound is mainly contained in the ethanolic root extract of *Mimosa pudica* L. [24].

In this study, most of the medicinal plants used to treat HT are of the herbaceous life form. This is similar to the situation for medicinal plants in southern Thailand [7]. From our literature review, it appears that the Tai Yuan is the ethnic group with the most reports of plants used for treating HT. As shown in Table 1, the number of plant species reported among the Tai Yuan and other ethnic groups tends to be inversely related to the number of studies cited for each ethnic group. However, the Tai Yuan, also known as Khon Mueang, make up the majority ethnic group whose population covers a large region of northern Thailand. Due to the large population size of the Tai Yuan, this ethnic group is most likely to be selected as the key informant for ethnobotanical studies. They maintain their livelihoods by relying on local plants.

Nowadays, technological progress increases while dependence on the natural resources of ethnic groups tends to decrease. In particular, knowledge concerning the groups tends to decrease, especially concerning their knowledge about medicinal plants. These plants have never produced medicine worldwide. Previously, they have only been reported in traditional medicine for HT treatment (in Thailand [25], Pakistan [26,27], China, and the Philippines [28]). These plants should be evaluated as new biotechnological sources of medicines for other diseases because for some plants, their pharmacological effects have never been reported. If these plants are proven effective in further pharmacological studies, it would be possible to discover bioactive compounds to promote their pharmaceutical production for the treatment of other diseases.

### 3.2. Use, Preparation, and Application of Plant Parts

In this study, all parts of the plants are used, including the leaves, underground parts, stems, infructescence, the entire plant, unspecified aerial parts, inflorescences, bark, seeds, galls, and exudate. Elsewhere, leaves have been commonly reported as being used in treatments for HT, such as in the Yala and Songkhla provinces in southern Thailand [7,8] and in Pakistan [26,27]. Leaves are often used for the extraction of medicine in traditional healing systems. They are soft and suitable for harvesting throughout all seasons, and their drug contents can be easily extracted [7].

In our results, decoction is the most used method of preparation for treating HT. It accounted for 49% of all use reports and is used for 131 species. Elsewhere, decoction is also commonly used to extract bioactive ingredients from medicinal plants and, in particular, for plants used to treat HT. This is true in the Songkhla and Yala provinces of southern Thailand [7,8], where traditionally licensed healers frequently use boiling for the preparation of medicine, and the same method is used in Pakistan [26,27].

Oral ingestion (as an internally used medicine and health food from chicken soup with herbs and vegetables) is the most common application method for treating HT among the 12 ethnic groups and accounts for 66% of all use reports and involves 165 species. Oral ingestion is the most used method by healers in both northern and southern Thailand. Oral ingestion is the only method of HT treatment by southern healers. Northern healers use several methods for HT treatment, such as oral ingestion (66%), herbal steaming (10%), bathing (8%), using a poultice (7%), inhalation (3%), using a compress (2%), and a liniment/a spray/wiping the body/blowing smoke into ears.

In addition, other applications of medicinal plants used for treating HT observed during our fieldwork, such as poultices and compresses (used as external medicine), are similar to those used in Thai traditional medicine [29].

### 3.3. Forms of Plants Used

Herbal recipes are the most commonly used form of medicine among the ethnic groups in northern Thailand and in Thai traditional medicine practice under the Ministry of Public Health of Thailand [7,8,30,31]. It is possible that the popularity of herbal recipes is based on cultural and historical inheritance, including knowledge about the process of how to use medicinal plants. This traditional knowledge was passed down from generation to generation for a long time until it finally became part of the local wisdom. The method of combining various herbs to enhance the drug activity of each of them and create synergistic effects for treating a disease is part of traditional medicine preparation [7,30].

In the Indigenous medicine of the Hmong, chicken soup is a health food that is used to support overall health [32], balance, and vitality. In interviews, some Hmong healers recommended various vegetables for the herbs in the chicken soup for HT patients. The herbs usually have aromatic characteristics (i.e., *Acorus gramineus* Aiton, *Elsholtzia penduliflora* W.W.Sm., or *Cymbopogon citratus* (DC.)) that are believed to recover the imbalance of blood circulation [6,7]. The Indigenous medicine knowledge of the Hmong healers related to specific herb selections for their HT patients is similar to the Indigenous medicine in Songkhla in southern Thailand [7]. Although health food cannot be classified as a drug, it can be considered a good strategy to promote health in a subtle way. However, health food is considered a part of biological-based therapy as nutritional care, which is a branch of alternative medicine that focuses on treating diseases using natural products for their health and wellbeing [33,34].

### 3.4. Important Plants Species for the Treatment of Hypertension in Northern Thailand

When the same plant is being used for the same medicinal purpose by many different ethnic groups, it has been taken as evidence that the used species could contain bioactive compounds that are relevant to its use in medicine [13]. This comparison of the agreement concerning uses between different cultures is an accepted way of highlighting bioactive medicinal plants. Independent discoveries by different ethnic groups suggest medical evidence for natural bioactivity. Accordingly, the top four medicinal plants with the highest FC and RCF values have already been reported for their bioactivity related to anti-hypertensive activity. The bioactive compounds found in these plants have been reported, e.g., the alkaloid L-mimosine [24].

*Blumea balsamifera* (L.) DC. (FC = 19, RFC = 0.1979, Supplementary Materials, Table S1, No. 50) is the most prominent species for treating HT among the ethnic communities in northern Thailand, and it has the highest agreement in terms of high use values with other ethnomedicinal research in Thailand [15]. It is also the plant with the highest degree of agreement in its uses in multiple villages of five different ethnic groups in northern Thailand (Hmong, Karen, Lisu, Tai Yai, and Tai Yuan). In other parts of the world, Chinese traditional medicine uses it to activate blood circulation. Wind-expelling and dampness dispersing in traditional medicine in the Philippines also use *Blumea balsamifera* (L.) DC to prepare a diuretic for the treatment of HT [28]. The bioactive compound from *B. balsamifera* leaves has been shown to inhibit the angiotensin-converting enzyme (ACE) in vitro, which is one of the mechanisms of anti-hypertensive activity [35]. However, the safety of short-term oil consumption using high doses (100% *w*/*v*) may lead to mild liver injury [36].

*Zingiber purpureum* Roscoe (FC = 13, RFC = 0.1354, Supplementary Materials, Table S1, No. 237) is used by four different ethnic groups (Hmong, Karen, Tai Yuan, and Tai Yai) to treat HT. For traditional medicinal treatments of HT, folk healers in Songkhla use the rhizome of *Zingiber purpureum* Roscoe, which they powder and boil as a medicine and use to stimulate the circular system [7]. The Thai traditional medicine system approved by Thailand’s Ministry of Public Health uses it in the same way [31]. For bioactive highlighting related to anti-HT activity, the aqueous extract showed anti-hypertensive activity with a decrease in mean arterial blood pressure, which was 3.54 times more active than the standard drug [5].

*Mimosa pudica* L. (FC = 12, RFC = 0.125 Supplementary Materials, Table S1, No. 111) is an exotic species (Table S1), and it has the highest frequency of citation in our study. It is used by five different ethnic groups (Karen, Khamu, Mien, Tai Yuan, and Tai Yai). The plant is widely used by local healers as one of the prominent medicinal species, as demonstrated in another ethnobotanical study in Thailand [22]. *Mimosa pudica* L. has already been investigated for its biological activity, confirming its anti-hypertension activity, which was recorded for diuretic activity by increasing urine volume and enhancing the elimination of sodium (Na+) and potassium (K+). The effect of the ethanolic root extract of *Mimosa pudica* L. may be related mainly to the alkaloid L-mimosine [24]. Certainly, each part of the plant can produce bioactive compounds in different concentrations. In the case of alkaloid L-mimosine, it was reported that it was extracted from the roots, but researchers did not evaluate the concentration level of the alkaloid L-mimosine in other parts of the plant [ 24]. In the future, it would be very useful to obtain phytochemical data about the concentration of this bioactive compound in the different parts of plants.

*Centella asiatica* (FC = 9, RFC = 0.0937, Supplementary Materials, Table S1, No. 28) is used by five different ethnic groups (Hmong, Karen, Lisu, Tai Yuan, and Tai Yai) in their traditional medicine system in HT treatment. The folk healers in southern Thailand use the leaves to reduce headaches and heart nourishment [8]. In the Thai traditional medicine system, the Ministry of Public Health uses *C. asiatica* for decreasing internal body heat [31]. *C. asiatica* is promoted as an antipyretic and anti-hypertensive herb [25], consumed as a local vegetable in Thailand’s healthcare system by the Department for Development of Thai Traditional and Alternative Medicine [25,37]. In existing research on anti-hypertension activity, *C. asiatica* has already been reported for its bioactive compounds [38].

From the data on plants used in traditional medicine for the treatment of HT, it appears that 60 out of the total 237 plant species used in Thailand’s northern region (Supplementary Materials, Table S1) are similar to the species listed for other regions in Thailand, including Songkhla [7], Yala [8], three southern border provinces [39], and hospitals under the office of the Permanent Secretary of the Ministry of Public Health [31]. We not only found data on the use of these plants in Thailand, but we also found similar use of the traditional medicinal plants in other Asian countries, such as in Iranian traditional medicine plants [40], Pakistani traditional medicine in South Asia [26,27], and Chinese traditional medicine in East Asia [41]. Additionally, we encountered data from traditional medicine in Africa, such as Nigerian traditional medicine [42], which was geographically remote and a very different culture. However, we did not find any information on the use of these plants to produce medicines worldwide in hypertension treatment. The remaining plants that have not been studied for anti-hypertension activity and toxicity should be further analyzed to confirm their efficacy.

In the part of the study on plants with pharmacological effects for treating HT, only the plants with an FC ≥ 2 and plants from different ethnicities were screened to further search for anti-hypertensive effects. We found 60 plant species from the data screening. We found 24 (40%) plant species that were reported to have an anti-hypertensive effect. These are *Aegle marmelos* (L.) Corrêa ex Roxb [43], *Allium sativum* L. [44], *Alpinia galanga* (L.) Willd [5], *Andrographis paniculata* (Burm.f.) Nees [45], *Biancaea sappan* (L.) Tod. [46], *Blumea balsamifera* (L.) DC. [28], *Centella asiatica* (L.) Urb. [38], *Chromolaena odorata* (L.) R.M.King & H.Rob. [47], *Citrus aurantiifolia aurantiifolia* (Christm.) Swingl [48], *Cymbopogon citratus* (DC.) Stapf [49], *Dendrophthoe pentandra* (L.) Miq. [50], *Gynostemma pentaphyllum* (Thunb.) Makino [51], *Mimosa pudica* L. [24], *Morinda angustifolia* Roxb. [52], *Moringa oleifera* Lam. [53], *Morus alba* L. [54], *Orthosiphon aristatus* (Blume) Miq. [55], *Oryza sativa* L. [56], *Phyllanthus acidus* (L.) Skeels [57], *Phyllanthus amarus* Schumach. & Thonn. [58], *Phyllanthus emblica* L. [59], *Piper nigrum* L. [60], *Saccharum officinarum* L. [61], *Tinospora crispa* (L.) Hook.f. & Thomson [62], and *Zingiber purpureum* Roscoe [5]. It appears that the anti-hypertensive effects of these plants consists of five different mechanisms, which are (1) a diuretic effect that removes the excess salt and fluid from the body; (2) as an angiotensin-converting enzyme inhibitor that blocks the angiotensin-converting enzyme of the renin–angiotensin system; (3) as a calcium channel blocker that resists large-vessel stiffness; (4) vasodilatation that relaxes the vascular wall; and (5) lowering of systolic and/or diastolic blood pressure.

Our results are from an inter-cultural or cross-cultural comparison based on the frequency of citation that reflects the degree of agreement in the use of medicinal plants. Therefore, it supports our hypothesis that “A medicinal plant that has a high frequency of citation and is the plant from the informants of different ethnicities, the medicinal plant is even more likely to have the anti-hypertensive effect”.

## 4. Materials and Methods

### 4.1. Data Sources

**Figure 2:**
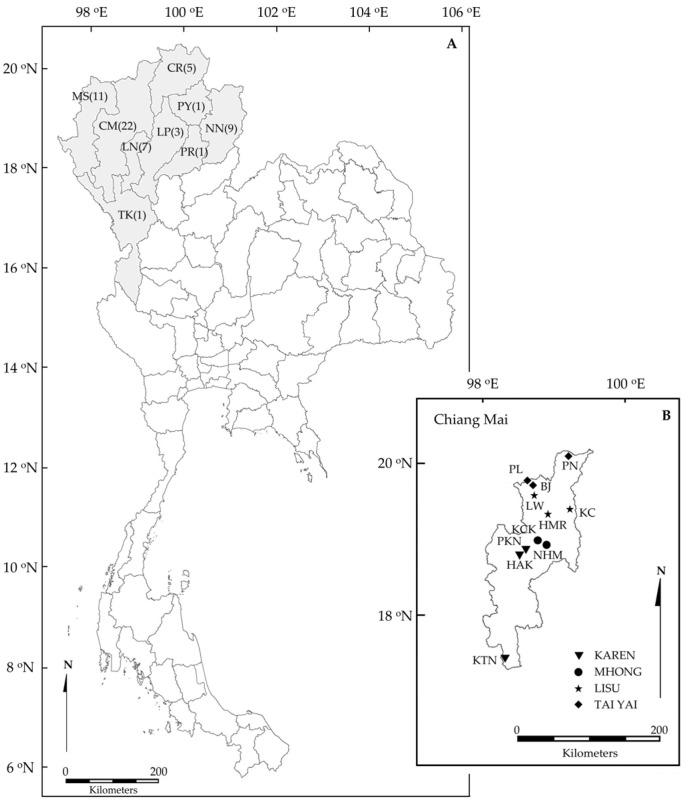
(**A**) The nine provinces providing ethnomedicinal plant knowledge for treating hypertension in this study (grey). The number after the abbreviation of the province name is the number of studies carried out in each province. (CM = Chiang Mai, CR = Chiang Rai, LP = Lampang, LN = Lamphun, MS = Mae Hong Son, NN = Nan, PY = Phayao, PR = Phrae, and TK = Tak). (**B**) Field observations in villages in Chiang Mai province (KAREN: Pha Ka Nok (PKN), Hoy Aee Kang (HAK), and Khun Tuen Noi (KTN); HMONG: Khun Chang Khian (KCK), Nhong Hoi Mai (NHM); LISU: Lea Wu (LW), Khun Chae (KC), and Hoy Ma Rid (HMR); TAI YAI: Piang Luang (PL), Pang Nai (PN), and Ban Jong (BJ).

The data for the ethnomedicinal plants used for treating HT were collected from both a literature review and a field survey. The voucher number shows the ethnomedicinal plant data from the field study by the collector (Table S1). The literature that was studied concerning ethnomedicinal plants used for treating HT in northern Thailand (Figure 2A) was selected by keywords, including “kwam dan” or “kwam dan sung” or “which means high blood pressure” in the Thai language. We used the following criteria: (1) plants should be recorded with their scientific names; (2) plants should also be recorded with their vernacular names; (3) data should be from medicinal books; and (4) data should be from journal articles, scientific reports, and theses published in both Thai and English. In this literature review, the information was extracted from 41 references published between 1987 and 2023. The journals and online theses were searched from the Thai Library Integrated System online database ‘’https://tdc.thailis.or.th/tdc/’ (accessed on 28 March 2025), which integrates theses from all universities in Thailand. Additionally, some unpublished data were extracted from theses in the Ethnobotany and Northern Thai Flora Laboratory, Department of Biology, Chiang Mai University, which is an ethnobotanical research unit [63] focused on northern Thailand. In order to avoid the duplication of sources by the same author based on the same file data, only original data were included (e.g., student thesis data that were subsequently published in journal articles).

### 4.2. Ethnobotanical Data Gathering

This paper presents medicinal plants used for treating the symptoms of hypertension from ethnic groups. It is knowledge that has been passed down from generation to generation (from the past to the present). The knowledge has a similar pattern despite the ethnic or cultural differences. Therefore, it is possible that these medicinal plants will contain active compounds for anti-hypertensive effects, leading to new discoveries.

The data from our field survey were collected from 41 healers in 11 villages in Chiang Mai province, as shown in Figure 2B, from December 2022 to November 2023. The key informant healers were selected because they had experiences in treating patients using herbalist skills, including herb collecting, planting, and keeping. As seen in the references from the past 10 years, the healer’s experience is a basic property that makes him/her accepted as a specialist [64,65]. As above, they were included in this study as key informants. The total number of healers was given by the chiefs. At least 80% of all healers were selected by a random sampling technique. This technique was used to avoid the intentional sampling of a healer. It secures the quality of sampling and reduces the risk of bias in the ethnobotanical study of medicinal plants, following previous research as guidelines [66]. In data collection on the treatment of HT from healers, we informed the healers before the interview about the role-play with a patient (we did not tell the healers that this patient had HT) who would tell them their symptoms (of HT). The healers were then interviewed about their medical practices when confronted with the patient’s symptoms (including additional history taking, physical examination, and the ethnic traditional medicinal diagnosis, respectively). After the healers made an ethnic traditional medicinal diagnosis, we interviewed them about the definition and causes of the diagnosed ethnic traditional medicine disease. The medical practices were recorded, and in particular, the names of the ethnic traditional medicine diseases were recorded in the local language. We also interviewed the healers about treatments, such as herbal medicine only, a massage combined with herbal medicine, etc. Then, the list of herbal plants (i.e., vernacular names, used parts, mode of preparation, and routes of administration) used for treating HT were recorded in this step. The role-playing (as a hypertensive patient) was carefully and precisely composed of the symptoms of HT patients by referencing medical journals [1,67,68] and textbooks [69] and tested by medical staff to evaluate the reliability that the symptoms indicated HT before the case study was used. We followed the research guidelines of Lumlerdkij and colleagues [70] and Neamsuvan and colleagues [7]. A local translator was available in case the key informants did not understand the Thai language.

### 4.3. Sample Collection and Identification

The vouchers of the plants were collected and identified by the authors using the taxonomic literature, including *Flora of Thailand*, *Flora of China*, *Flora of Java*, and *Thai Forest Bulletin*. Each plant species was surveyed during the fruiting and flowering seasons by following the procedure of plant collection and preservation relating to the ethnobotanical method described in *A Field Guide to Forest Trees of Northern Thailand* [71]. The voucher specimens were deposited at the Queen Sirikit Botanic Garden Herbarium (QBG), Chiang Mai, Thailand. A few species that are well-known cultivated plants were not vouchered, e.g., the garlic (*Allium sativum* L.), galangal (*Alpinia galanga* (L.) Willd.), lemongrass (*Cymbopogon citratus* (DC.) Stapf), and sugarcane (*Saccharum officinarum* L.).

### 4.4. Data Validation and Organization

The scientific names of plants were checked against the World Checklist of Vascular Plants “https://powo.science.kew.org” (accessed on 28 March 2025). Tentative scientific names of plants, which were extracted from books, were identified in the Thai medicinal plants standard database (i.e., Thai Herbal Pharmacopoeia “https://bdn.go.th/thp/home”(accessed on 28 March 2025), *Manual of Herbal Medicine Production Standard* [72], and an explanation of King Narai Remedies [73]). For example, data from books [74] were extracted from the Library of Pharmacy, Department of Pharmacy, Chiang Mai University, which is the repository for the translation and interpretation of the Lanna–Thai medicinal plant research in northern Thailand. The books describe many medicinal herbs presented as transliterations of herbal medicine recipes that appeared in palm leaf scripture and mulberry-pulp booklets by Lanna folk healers.

Information about the parts of the plants used was derived from the references and classified into bark, bulbil, the entire plant, exudate, leaves (including young shoots and shoots), inflorescences (including style and peduncle), infructescence, seeds and stems, unspecified aerial parts, and underground parts (including bulb, roots, and rhizome). Methods of preparation and routes of administration were derived from the original reports (literature-based data) and specific uses of local healers’ perceptions (field-based data). Following the *Economic Botany Data Collection Standard* [22] and general ancient medicine textbooks in Thai pharmacy [75], the preparation data were categorized and grouped into burning, cooking, crushing, decoction, drying, fresh, grinding, infusion, pill, pounding, powdering, and soaking. The medicinal application data were categorized and grouped into bath, blowing smoke into ears, compress, fumigate, herbal steam, inhalation, liniment, oral ingestion, poultice, spray, and wiping the body. The plant life forms or habits were derived from the references, including the literature of the Thai Plant Databases, updated “https://botany.dnp.go.th/mplant/index.html” (accessed on 28 March 2025) and grouped into herb, shrub, climber, tree, grass, fern, and lichen. For the forms of plant use, the data were categorized by their ethnomedicinal use and divided into three groups: (1) a medicinal plant recipe means a medicinal preparation consisting of two or more plants, and they may be mixed with non-plant ingredients (honey and salt); (2) single-use means the use of only one plant species, but it may be associated with other non-plant ingredients; and (3) food, which means that the plants were cooked to make healthy food.

### 4.5. Data Analyses

#### 4.5.1. Frequency of Citation (FC)

The frequency of citation is the percentage of informants who mention the species for a given use [7]. To evaluate the degree of agreement among informants who use the medicinal plant for treating HT, we used the term frequency of citation [76]. The total citation of each species estimates the importance of the medicinal plants. Plants with a high degree of agreement concerning the same use were thought to potentially have high bioactive effects [13].

Here, the number of informants who mentioned the species was derived from both a literature review and a field survey. For data from the literature review where we only knew the ethnic minority and not the individual informants interviewed, we defined a “pseudo-informant”, representing an ethnicity as a reference in our analysis. For example, in Sumridpiem [77], the ethnobotany of medicinal plants in Lamphun province was studied among the different ethnic groups “Tai Yong” and “Tai Yuan”, who counted as two pseudo-informants. Also, the ethnobotany of medicinal plants in only one ethnic group counted as two pseudo-informants when we knew about the province for each informant. For example, in Brun and Schmacher [6], the ethnobotany of medicinal plants used by the “Tai Yuan” in Lamphun and Chiang Mai provinces was studied; therefore, the “Tai Yuan” were counted as two pseudo-informants.

In our study, the number of informants who mentioned the species was derived from both the literature review (55 pseudo-informants) and the field survey (41 local healers). The total number (n) of 96 informants was used for the calculations in our analysis.

#### 4.5.2. Relative Frequency of Citation (RFC)

The relative frequency of citation was calculated to determine the level of traditional knowledge concerning the use of medicinal plants in the study area [27]. This index shows the local importance of each plant species used among the informants who cited that species [14,78]:

RFC = FC/N

where the FC is the number of informants who mention the use of the species, and N is the total number of informants who participated in the study [76].

#### 4.5.3. Use Reports (URs)

Use reports (URs) are the number of reports mentioned by pseudo-informants from the literature review and informants from the fieldwork. The use of the same medicinal plant by informants from different ethnic groups suggests that the plant could be pharmacologically active [13,76].

We defined a “plant use” for a given species as use associated with a unique combination of use states with a specific ethnicity, part of plant used, method of preparation, and application. The data from the literature review where we only knew the ethnic minority and not the individual informants interviewed, we counted as pseudo-informants to represent an ethnicity as a reference in our analysis. For example, the medicinal plant species number 202 (Supplementary Table S1, *Melicope pteleifolia* (Champ. ex Benth.) T.G. Hartley) was reported by Inta and colleagues [79], and the ethnobotany of the medicinal plants in Mae Hong Son province was mentioned by two different ethnic groups “Lua” and “Tai Yuan”, who counted as two pseudo-informants. Meanwhile, the data from the fieldwork of the medicinal plant species number 202 were mentioned by one informant in Lisu, so it counted as one informant. Per the above statements, there was a total of three informants from three different ethnicities who mentioned the medicinal plant species number 202. The root of the medicinal species was decocted and ingested orally by two pseudo-informants (the Tai Yuan and the Lua). Also, only the leaves of the medicinal species were decocted and steamed by another informant (the Lisu); therefore, it was counted as three use reports (URs) for this species.

## 5. Conclusions

From our field data and literature review, we compiled ethnobotanical knowledge about plants used for treating HT. We found 237 medicinal plant species belonging to 194 genera and 82 families. The most important species is *Blumea balsamifera* (L.) DC., which has the highest values for both frequency of citation (FC) and relative frequency of citation (RFC), while the most important plant family is Fabaceae.

Our review could lead to the discovery of alternative medicines for treating HT. However, the possibility of toxicity effects in these plants has been reported (e.g., mild liver injury). The potential of secondary or toxicity reactions of medicinal plants could be regulated by the method and the specific plant parts used. Future investigations of phytochemical compounds and pharmacological research are needed to confirm the efficacy of treatments. The plant part used and the preparation method could play a critical role in the volume of their bioactive compounds.

Finally, in addition to medicinal information, this review emphasizes the importance of traditional knowledge and the significance of knowledge management systems to create academic materials and public awareness campaigns that provide accurate information about herbal medicines. Their benefits and potential risks can be integrated into policy planning in herbal medicine research and guidelines for treating HT.

## Figures and Tables

**Table 1:** Number of species and use reports of medicinal plants used for treating hypertension by 12 ethnic groups in northern Thailand.

Ethnic Group	Language Family			DATA		Species	Use Reports	References in Literature Review (Supplementary Materials, Table S2)
		Literature Review		Field Survey				
		Species	Use Reports	Species	Use Reports			
Akha	Sino-Tibetan	5	5	-	-	5	5	[2,30]
Hmong	Hmong–Mien	17	20	34	93	50	113	[2,5,22,29,33]
H’Tin	Austroasiatic	1	1	-	-	1	1	[24]
Karen	Sino-Tibetan	39	47	24	47	55	92	[2,8–10,13,16, 17,25,35–37,41]
Khamu	Austroasiatic	5	5	-	-	5	5	[33]
Lisu	Sino-Tibetan	4	4	19	56	22	60	[15,23,25]
Lua	Austroasiatic	7	7	-	-	7	7	[13,33]
Mien	Hmong–Mien	8	11	-	-	8	11	[26,33,38]
Tai Lue	Tai–Kadai	5	6	-	-	5	6	[21,38]
Tai Yai	Tai–Kadai	8	13	52	109	52	122	[1,11,13,27,40]
Tai Yong	Tai–Kadai	8	10	-	-	8	10	[34]
Tai Yuan	Tai–Kadai	121	206	-	-	121	206	[3,4,7,12,14,18,19,21, 25,28,31,32,34,39]
	Total	173	339	96	320	237	659	[41]

**Table 2:** Plant families and number of species that are used for treating hypertension among 12 ethnic groups in northern Thailand, indicating the number of species in each family and their percentage of the total number of species.

Families	Species (%)	Families	Species (%)	Families	Species (%)
Fabaceae	23 (10)	Hypoxidaceae	2 (<1)	Loranthaceae	1 (<1)
Asteraceae	17 (7)	Lauraceae	2 (<1)	Lythraceae	1 (<1)
Acanthaceae	14 (6)	Malvaceae	2 (<1)	Marantaceae	1 (<1)
Lamiaceae	12 (5)	Melastomataceae	2 (<1)	Marsileaceae	1 (<1)
Zingiberaceae	10 (5)	Musaceae	2 (<1)	Meliaceae	1 (<1)
Apiaceae	8 (4)	Pinaceae	2 (<1)	Moringaceae	1 (<1)
Piperaceae	7 (3)	Plantaginaceae	2 (<1)	Myrtaceae	1 (<1)
Poaceae	7 (3)	Plumbaginaceae	2 (<1)	Nelumbonaceae	1 (<1)
Rutaceae	7 (3)	Rhamnaceae	2 (<1)	Oleaceae	1 (<1)
Phyllanthaceae	6 (3)	Smilacaceae	2 (<1)	Ophioglossaceae	1 (<1)
Solanaceae	6 (3)	Urticaceae	2 (<1)	Oxalidaceae	1 (<1)
Apocynaceae	5 (2)	Verbenaceae	2 (<1)	Pandanaceae	1 (<1)
Commelinaceae	4 (2)	Annonaceae	1 (<1)	Passifloraceae	1 (<1)
Euphorbiaceae	4 (2)	Araceae	1 (<1)	Polygalaceae	1 (<1)
Moraceae	4 (2)	Arecaceae	1 (<1)	Polypodiaceae	1 (<1)
Amaryllidaceae	3 (1)	Asparagaceae	1 (<1)	Ranunculaceae	1 (<1)
Amaranthaceae	3 (1)	Betulaceae	1 (<1)	Salicaceae	1 (<1)
Bignoniaceae	3 (1)	Boraginaceae	1 (<1)	Salvadoraceae	1 (<1)
Capparaceae	3 (1)	Bromeliaceae	1 (<1)	Santalaceae	1 (<1)
Cucurbitaceae	3 (1)	Calophyllaceae	1 (<1)	Sapindaceae	1 (<1)
Menispermaceae	3 (1)	Campanulaceae	1 (<1)	Sapotaceae	1 (<1)
Rubiaceae	3 (1)	Cardiopteridaceae	1 (<1)	Saururaceae	1 (<1)
Acoraceae	2 (<1)	Caricaceae	1 (<1)	Scrophulariaceae	1 (<1)
Anacardiaceae	2 (<1)	Crassulaceae	1 (<1)	Usneaceae	1 (<1)
Araliaceae	2 (<1)	Cyperaceae	1 (<1)	Viburnaceae	1 (<1)
Basellaceae	2 (<1)	Dioscoreaceae	1 (<1)	Vitaceae	1 (<1)
Brassicaceae	2 (<1)	Ebenaceae	1 (<1)		
Combretaceae	2 (<1)	Lecythidaceae	1 (<1)		

**Table 3:** Mode of preparation of medicinal plant species used for treating hypertension by 12 ethnic groups in northern Thailand.

Mode of Preparation	Description Method	Use Reports (%)
Decoction	Boiling water with single fresh/dried material or combining with others (herbs or sugar)	326 (49)
Fresh	No preparation	74 (11)
Burning	Heating the fresh/dried materials until soft; pounding into rough materials and heating; burning into ash and powdering	48 (7)
Cooking	Cooking the materials as a food (i.e., chicken soup)	39 (5)
Pounding	Pounding only the materials; mixing the pounded materials with sugar and/or water; mixing the pounded materials with cow dung	26 (4)
Infusion	Infusing the materials in a container of salt water/honey/alcohol for weeks/months	22 (3)
Powdering	Powdering the dried materials; roasting the materials until golden brown before powdering and mixing with boiled water or other herbs	20 (3)
Grinding	Grinding the dried materials with a little of water and adding the grinded materials into a cup of hot water	15 (2)
Soaking	Soaking the fresh/dried materials in a container of hot water /water at room temperature for a while	14 (2)
Crushing	Pounding into fine materials and squeezing the pounded material for juice with/without adding a teaspoon of honey	12 (2)
Pill	Natural drying and powdering the materials before filling into a capsule or combining with others before rolling into a bolus	3 (<1)
Drying	Natural drying of the materials until crispy	2 (<1)

**Table 4:** Number of use reports and % for medicinal application of plants used for treating hypertension among 12 ethnic groups in northern Thailand.

Application	Use Reports (%)
Oral ingestion	399 (66)
Herbal steam	60 (10)
Bath	48 (8)
Poultice	42 (7)
Inhalation	19 (3)
Compress	12 (2)
Liniment	9 (1)
Spray	8 (1)
Wiping the body	1 (<1)
Blowing smoke into ears	1 (<1)
Fumigate	1 (<1)

**Table 5:** Number of use reports concerning the form of plant used for treating hypertension among 12 ethnic groups in northern Thailand.

Ethnic Group	Form of Plant Use (Use Reports)					
	Herbal Recipes		Single Herbs		Food	
	Use Reports	Species	Use Reports	Species	Use Reports	Species
Akha	1	1	3	3	-	-
Hmong	11	7	89	36	30	15
H’Tin	1	1	-	-	-	-
Karen	24	18	61	42	7	3
Khamu	-	-	3	3	2	2
Lisu	52	16	10	10	-	-
Lua	2	2	4	4	1	1
Mien	-	-	7	4	4	4
Tai Lue	3	3	3	2	-	-
Tai Yai	107	43	14	9	-	-
Tai Yong	2	2	5	4	3	2
Tai Yuan	88	72	109	61	9	8
Total	292	130	305	122	56	29

## Data Availability

Data are contained within the article and supplementary materials.

## Supplementary Materials

The following supporting information can be downloaded at: https://www.mdpi.com/article/10.3390/plants14071066/s1, Table S1: Medicinal plants and uses for treatment of hypertension in northern Thailand from both primary data (field study by authors) and secondary data, Table S2: Details of the 41 references used to analyze northern Thai hypertension plant data.

## References

[1] Thai Hypertension Society (2019). Thai Guidelines on The Treatment of Hypertension 2019. https://www.thaihypertension.org/guideline.html.

[2] Thai Ministry of Public Health and World Health Organization (2019). Hypertension Care in Thailand: Best Practices and Challenges. https://iris.who.int/bitstream/handle/10665/330488/9789290227403-eng.pdf.

[3] Buranakitjaroen P., Sriussadaporn S., Phoojaroenchanachai M., Sangrasert P., Saravich S. (2003). Angiotensin converting enzyme inhibitor induced cough: Experience in Siriraj Hospital. J. Med. Assoc. Thai..

[4] Chobanian A.V., Bakris G.L., Black H.R., Cushman W.C., Green L.A., Izzo J.L., Jones D.W., Materson B.J., Oparil S., Wright J.T. (2003). The Seventh Report of the Joint National Committee on Prevention, Detection, Evaluation, and Treatment of High Blood Pressure: The JNC 7 Report. JAMA.

[5] Manosroi A., Lohcharoenkal W., Khonsung P., Manosroi W., Manosroi J. (2013). Potent antihypertensive activity of Thai-Lanna medicinal plants and recipes from MANOSROI III database. J. Pharm Bio..

[6] Brun V., Schmacher T. (1994). Traditional Herbal Medicine in Northern Thailand.

[7] Neamsuvan O., Komonhiran P., Boonming K. (2018). Medicinal plants used for hypertension treatment by folk healers in Songkhla province, Thailand. J. Ethnopharmacol..

[8] Hookheaw S., Neamsuvan O. (2020). Ethnopharmacology in Treatment of Hypertension from Yala Province, Thailand. SWU Sci. J..

[9] Phumthum M., Balslev H. (2019). Use of Medicinal Plants Among Thai Ethnic Groups: A Comparison. J. Econ. Bot..

[10] Srithi K., Trisonthi C., Wangpakapattanawong P., Srisanga P., Balslev H. (2012). Plant Diversity in Hmong and Mien Homegardens in Northern Thailand. J. Econ. Bot..

[11] Inta A., Kampuansai J., Kutanan W., Srikummool M., Pongamornkul W., Srisanga P., Panyadee P. (2023). Women’s wellness in the mountains: An exploration of medicinal plants among tibeto-burman groups in Thailand. Heliyon.

[12] Tangjitman K., Wongsawad C., Kamwong K., Sukkho T., Trisonthi C. (2015). Ethnomedicinal plants used for digestive system disorders by the Karen of nortern Thailand. J. Ethnobiol. Ethnomed..

[13] Srithi K., Trisonthi C., Inta A., Balslev H. (2019). Cross-cultural Comparison of Medicinal Plants Used to Treat Infections in Northern Thailand. J. Econ. Bot..

[14] Kantasrila R., Pandith H., Balslev H., Wangpakapattanawong P., Panyadee P., Inta A. (2020). Medicinal Plants for Treating Musculoskeletal Disorders among Karen in Thailand. Plants.

[15] Srithi K. (2012). Comparative Ethnobotany in Nan Province, Thailand. Ph.D. Thesis.

[16] Panyadee P., Balslev H., Wangpakapattanawong P., Inta A. (2019). Medicinal plants in homegardens of four ethnic groups in Thailand. J. Ethnopharmacol..

[17] Phumthum M., Srithi K., Inta A., Junsongduang A., Tangjitman K., Pongamornkul W., Trisonthi C., Balslev H. (2018). Ethnomedicinal plant diversity in Thailand. J. Ethnopharmacol..

[18] Panyadee P., Wangpakapattanawong P., Inta A., Balslev H. (2023). Very High Food Plant Diversity among Ethnic Groups in Northern Thailand. Diversity.

[19] Hl K., Sl S., Ps V., Vh P., Am S., Sibgatullah M. (2015). Diuretic Activity of Ethanolic Root Extract of Mimosa Pudica in Albino Rats. J. Clin. Diagn. Res..

[20] Marles R.J., Farnsworth N.R. (1995). Antidiabetic plants and their active constituents. Phytomedicine.

[21] Ahmad L., Semotiuk A., Zafar M., Ahmad M., Sultana S., Liu Q.R., Zada M.P., Abidin S.Z., Yaseen G. (2015). Ethnopharmacological documentation of medicinal plants used for hypertension among the local communities of DIR Lower, Pakistan. J. Ethnopharmacol..

[22] Malik K., Ahmad M., Bussmann R.W., Tariq A., Ullah R., Alqahtani A.S., Shahat A.A., Rashid N., Zafar M., Sultana S. (2018). Ethnobotany of Anti-hypertensive Plants Used in Northern Pakistan. Front. Pharmacol..

[23] (2015). Thai Traditional Medicine Knowledge Fun.

[24] Saising J., Maneenoon K., Sakulkeo O., Limsuwan S., Götz F., Voravuthikunchai S.P. (2022). Ethnomedicinal Plants in Herbal Remedies Used for Treatment of Skin Diseases by Traditional Healers in Songkhla Province, Thailand. Plants.

[25] Khuankaew S., Srithi K., Tiansawat P., Jampeetong A., Inta A., Wangpakapattanawong P. (2014). Ethnobotanical study of medicinal plants used by Tai Yai in Northern Thailand. J. Ethnopharmacol..

[26] Bunpean A., Subhadhirasakul S., Neamsuvan O., Siriyong T. (2021). The use of herbal medicine to treat hypertension in hospitals under the office of the Permanent Secretary of the Ministry of Public Health. J. Med. Health Sci..

[27] Kathy D. (2007). Elder abuse in context of poverty and deprivation and emergency department care. Aust. Emerg. Nurs. J..

[28] Nguanchoo V., Prachaya S., Swangpol S., Prathanturarug S., Jenjittikul T. (2014). Food plants in Hmong cuisine in Northern Thailand. Thai J. Bot..

[29] Institute of Medicine (US) Committee on the Use of Complementary and Alternative Medicine by the American Public (2005). Complementary and Alternative Medicine in the United States. https://www.ncbi.nlm.nih.gov/books/NBK83804/.

[30] Pang Y., Wang D., Fan Z., Chen X., Yu F., Hu X., Wang K., Yuan L. (2014). *Blumea balsamifera*—A phytochemical and pharmacological review. Molecules.

[31] See G.L.L., Arce F.V., Deliman Y.C. (2016). ACE (angiotensin converting enzyme) inhibition activity of oven–dried and air–dried Sambong *Blumea balsamifera* L. (dc.) tea. Int. J. Pharmacogn. Phytochem. Res..

[32] Pang Y.X., Fan Z.W., Wang D., Yang Q., Wang K., Chen X.L. (2014). External application of the volatile oil from *Blumea balsamifera* may be safe for liver—A study on its chemical composition and hepatotoxicity. Molecules.

[33] Department for Development of Thai Traditional and Alternative Medicine (2019). Guide Line for Taking Care of High Blood Pressure Patients with Integrated Medicine. https://thaicam.dtam.moph.go.th/wp-content/uploads/2019/06/คู่มือดูแลความดัน.pdf.

[34] (2019). Food and Drug Administration National List of Essential Drugs. https://ratchakitcha.soc.go.th/documents/140D130S0000000004500.pdf.

[35] Harwoko P.S., Nugroho A.E. (2014). Triterpenoid-rich Fraction of *Centella asiatica* Leaves and In Vivo Antihypertensive Activity. Int. Food Res. J..

[36] Sangmanee R. (2017). Using Local Wisdom with Self-care Behavior of Hypertension Patients in the Three Southern Border Provinces. Prin. Narad. Uni. J..

[37] Baharvand A.B., Asadi S.M. (2017). Medicinal plants and treatment of hypertension; evidence from Iran. J. Nephropharm..

[38] Xingjiang X., Pengqian W., Lian D., Wei L., Fuyong C., Shengjie L., Xiaoke L., Kelei S., Hu Y., Yanwei X. (2019). Efficacy and safety of Chinese herbal medicine Xiao Yao San in hypertension: A systematic review and meta-analysis. Phytomedicine.

[39] Sonibare A.M., Abegunde B.R. (2012). Ethnobotanical study of medicinal plants used by the Laniba village people in South Western Nigeria. Afr. J. Microbiol. Res..

[40] Ghelani H.S., Patel B.M., Gokani R.H., Rachchh M.A. (2014). Evaluation of polyherbal formulation for antihypertensive activity in albino rats. J. Ayu..

[41] Al-Qattan K.K., Alnaqeeb M.A., Ali M. (1999). The antihypertensive effect of garlic (*Allium sativum*) in the rat two-kidney–one-clip Goldblatt model. J. Ethnopharmacol..

[42] Zhang C., Tan B. (1996). Hypotensive activity of aqueos extract of Andrographis paniculata in rat. Clin. Exp. Pharmacol. Physiol..

[43] Hu C.M., Kang J.J., Lee C.C., Li C.H., Liao J.W., Cheng Y.W. (2003). Induction of vasorelaxation through activation of nitric oxide synthase in endothelial cells by brazilin. Eur. J. Pharmacol..

[44] Ikewuchi J.C., Ikewuchi C.C., Enuneku E.C., Ihunwo S.A., Osayande O.I., Batubo D.B., Manuel D.I. (2012). Alteration of Blood pressure indices and pulse rates by an aqueous extract of the leaves of *Chroolaena odorata* (L) King and Robinson (Asteraceae). Pac. J. Sci. Technol..

[45] Al-Snafi A.E. (2017). Medicinal plants for prevention and treatment of cardiovascular diseases—A review. J. Pharm..

[46] Carbajal D., Casaco A., Arruzazabala L., Gonzalez R., Tolon Z. (1989). Pharmacological study of *Cymbopogon citratus* leaves. J. Ethnopharmacol..

[47] Sjakoer N.A.A., Mubarakati N.J., Taufiq A. (2021). Investigation of Excellent ACE Inhibitor Agents from *Scurrula atropurpure* and *Dendrophthoe pentandra* for Anti-Hypertension. Chiang Mai University. J. Nat. Sci..

[48] Mishra R.N., Joshi D. (2011). Jiao Gu Lan (*Gynostemma pentaphyllum*): The Chinese Rasayan-Current Research Scenario. Int. J. Pharm. Res. Bio-Sci..

[49] Xiang W., Song Q.S., Zhang H.J., Guo S.P. (2008). Antimicrobial anthraquinones from *Morinda angustifolia*. Fitoterapia.

[50] Dangi S.Y., Jolly C.I., Narayanan S. (2002). Antihypertensive activity of the total alkaloids from the leaves of *Moringa oleifera*. Pharm. Biol..

[51] Dytha A.D., Sri M. (2018). Influence of mulberry leaf extract (*Morus alba* L.) on diuretic activity of male white wistar strain rat. Drug Invent. Today.

[52] Ohashi K., Bohgaki T., Shibuya H. (2000). Antihypertensive substance in the leaves of kumis kucing (*Orthosiphon aristatus*) in Java Island. J. Pharm. Soc. Jpn..

[53] Shobako N., Ohinata K. (2020). Anti-Hypertensive Effects of Peptides Derived from Rice Bran Protein. Nutrients.

[54] Leeya Y., Mulvany M.J., Queiroz E.F., Marston A., Hostettmann K., Jansakul C. (2010). Hypotensive activity of an n-butanol extract and their purified compounds from leaves of *Phyllanthus acidus* (L.) Skeels in rats. Eur. J. Pharmacol..

[55] Srividya N., Periwal S. (1995). Diuretic, hypotensive and hypoglycaemic effect of *Phyllanthus amarus*. Indian J. Exp. Biol..

[56] Bhatia J., Tabassum F., Sharma A.K., Bharti S., Golechha M., Joshi S. (2011). *Emblica officinalis* exerts antihypertensive effect in a rat model of DOCA-salt-induced hypertension: Role of (p) eNOS, NO and oxidative stress. Cardiovas. Toxicol..

[57] Taqvi S.I.H., Shah A.J., Gilani A.H. (2008). Blood pressure lowering and vasomodulator effects of piperine. J. Cardiovas. Pharmacol..

[58] Wczassek L.R., Carlin S.D., Landell M.G.D., Gamberini M.T. (2022). Dopaminergic, cholinergic and nitrinergic pathways implicated in blood pressure lowering effects of *Saccharum officinarum* L. (Sugarcane) on rats. Phytomed. Plus.

[59] Praman S., Mulvany M.J., Allenbach Y., Marston A., Hostettmann K., Sirirugsa P., Jansakul C. (2011). Effects of an n-butanol extract from the stem of *Tinospora crispa* on blood pressure and heart rate in anesthetized rats. J. Ethnopharmacol..

[60] Trisonthi C., Trisonthi P., Srisanga P., Inta A. (2018). Ethnobotany: A Science of Folk Wisdom.

[61] Department of Social Development and Welfare (2016). Highland Communities Within 20 Provinces of Thailand.

[62] Young G. (1962). The Hill Tribes of Northern Thailand.

[63] Dessaint Y.W. (1981). The Tin (Mal) dry rice cultivators of northern Thailand and northern Laos. J. Siam Soc..

[64] Kulsomboon S., Ubonkhaw P. (2011). A Guide to Assessing of Folk Healer.

[65] Notification of the Ministry of Health: Certification of Folk Healer B.E. 2562. Government Gazette. Rule Number 136. Special Section Number 146 ngor. Pages1–13. http://www.ratchakitcha.soc.go.th/DATA/PDF/2562/E/146/T_0001.PDF?fbclid=IwAR33dXLdOv93zyzCXKCGQkCyWmOw7C-IpJ0oG3DNpIaAWO8WVpT1AJBSaiw.

[66] Medeiros P.M., Ladio A.H., Albuquerque U.P. (2014). Sampling problems in Brazilian research: A critical evaluation of studies on medicinal plants. Rev. Bras. Farmacogn..

[67] Gulzar A., Sadeeqa S. (2019). Hypertension: A Case Study. J. Virol. Immun..

[68] Lunganu A., Weiss E.A., Balahura A.M., Bartos D., Badila E.A. (2019). Long Forgotten Diagnosis–A Case of Malignant Hypertension. J. Hypertens..

[69] Achanuprap S. (2008). Examination and Treatment of General Disease Textbook 2.

[70] Lumlerdkij N., Tantiwongse J., Booranasubkajorn S., Boonrak R., Akarasereenont P., Laohapand T., Heinrich M. (2018). Understanding cancer and its treatment in Thai traditional medicine: An ethnopharmacological-anthropological investigation. J. Ethnopharmacol..

[71] Gardner S., Sidisunthorn P., Anusarnsunthor V. (2007). A Field Guide to Forest Trees of Northern Thailand.

[72] National Drug System Development Committee (2017). Manual Book for the Production and Quality Assurance of Medicinal Herbal Hospital Formulas in the National List of Essential Drugs National List of Essential Medicines 2016.

[73] Picheansoonthon C., Chawalit M., Jiravongse V. (2017). An Explanation of King Narai Remedies: The Special Edition Commemorated the King 72nd Birthday Anniversary (5 December 1999).

[74] Manosroi J., Manosroi A. (1994). Lanna Pharmacy: Lanna Medicinal Plants Recipes.

[75] Department of Thai Traditional and Alternative Medicine, Ministry of Public Health (1998). General Ancient Medicine Textbooks: Thai Pharmacy.

[76] Tardío J., Santayana P.D.M. (2008). Cultural importance indices: A comparative analysis based on the useful wild plants of Southern Cantabria. J. Econ. Taxon. Bot..

[77] Sumridpiem P. (2018). Utilization Analysis of Medicinal Plants Between Tai Yong and Tai Yuan in Lamphun Province. Master’s Thesis.

[78] Vitalini S., Iriti M., Puricelli C., Ciuchi D., Segale A., Fico G. (2013). Traditional knowledge on medicinal and food plants used in Val San Giacomo (Sondrio, Italy)—An alpine ethnobotanical study. J. Ethnopharmacol..

[79] Inta A., Srisanga P., Pongamornkul W., Khongkeaw B., Muangmun N., Muangmun W. (2020). The Biological Resources Inventory of Medicinal Plants and Folk Wisdom in Mae Hong Son and Lamphune Province.

