# Supplementary material for: Plants Used for Treating Hypertension Among Ethnic Groups in Northern Thailand
Source: Plants (Basel). 2025 Mar 30;14(7):1066. doi: 10.3390/plants14071066 (PMC11991033; doi:10.3390/plants14071066)
Supplement: Supplementary file 1 [file plants-14-01066-s001.zip › plants-3487228-supplementary.pdf]

Supplementary Materials

Table S1. Medicinal plants and uses for treatment of hypertension in northern Thailand from both primary data (field study by authors) and secondary data.

Scientific Name	Habit	Part	Preparation	Application	Type of medicine	Use reports	FC	RFC	Study area (Province)	Ethnic (References)/Voucher number
**Acanthaceae**										
1) *Acanthus ebracteatus* Vahl	Shrub	Leaves	Decoction	Oral ingestion	Recipes	1	1	0.0104	CRI	Tai Yuan (Tovaranonte, 2002)
2) *Andrographis paniculata* (Burm.f.) Nees [a,b]	Herb	Leaves<br>Leaves<br>Non-aerial parts<br><br><br>Leaves<br>Leaves<br>Non-aerial parts	Decoction<br>Decoction,<br>Soaking,<br>Powdering<br>Pill, Decoction<br>Decoction<br>Decoction<br>Decoction	Oral ingestion<br>Oral ingestion<br>Oral ingestion<br><br><br>Oral ingestion<br>Oral ingestion<br>Oral ingestion,<br>Herbal steam<br>~~stream,~~ Bath	Single used<br>Single used<br>Recipes<br><br><br>Single used<br>Recipes<br>Recipes	14	7	0.0729	LPN<br>LPN<br>LPN<br><br><br>PRE<br>CRI<br>CMI	Tai Yuan (Inta et al., 2013B)<br>Tai Yong (Sumridpiem, 2017)<br>Tai Yuan (Sumridpiem, 2017)<br><br><br>Tai Yuan (Inta et al., 2019)<br>Tai Yuan (Tovaranonte, 2002)<br>P. Sumridpiem 2023 12/ Tai Yai (N=2)
3) *Asystasia gangetica* (L.) T. Anderson	Herb	Non-aerial parts	Pounding	Oral ingestion	Recipes	1	1	0.0104	LPN	Tai Yuan (Sumridpiem, 2017)
4) *Barleria lupulina* Lindl. [a]	Shrub*	Leaves	Pounding	Oral ingestion	Recipes	1	1	0.0104	LPN	Tai Yong (Sumridpiem, 2017)
5) *Clinacanthus nutans* (Burm.f.) Lindau	Climber	Leaves<br>Leaves	Pounding<br>Pounding	Oral ingestion<br>Oral ingestion	Recipes<br>Recipes	2	2	0.0208	LPN<br>LPN	Tai Yong (Sumridpiem, 2017)<br>Tai Yuan (Sumridpiem, 2017)
6) *Justicia gendarussa* Burm.f.	Shrub*	Entire plant	Decoction	Oral ingestion	Single used	1	1	0.0104	CMI	Tai Yuan (Brun and Schmacher, 1994)
7) *Nicoteba betonica* (L.) Lindau	Shrub*	Stems, Roots	Grinding	Oral ingestion	Recipes	2	1	0.0208	CMI	P. Sumridpiem 2022 43/Tai Yai (N=1)
8) *Phlogacanthus curviflorus* (Nees) Nees	Shrub	Leaves	Decoction	Herbal steam	Recipes	1	1	0.0104	CMI	P. Sumridpiem 2022 66/Tai Yai (N=1)
9) *Pseuderanthemum* lanceophyllum Choopan	Shrub	Leaves,<br>Young shoot<br>Leaves<br>Leaves<br>Leaves	Decoction,<br>Fresh<br>Fresh<br>Fresh<br>Fresh	Oral ingestion<br>Oral ingestion<br>Oral ingestion<br>Oral ingestion<br>Oral ingestion	Single used<br>Single used<br>Food<br>Food<br>Food	5	4	0.0416	CMI<br><br>LPN<br>NAN<br>NAN	Tai Lue (Muangyen, 2013)<br><br>Tai Yuan (Sumridpiem, 2017)<br>Khamu (Srithi, 2012)<br>Mien (Srithi, 2012)
10) *Rhinacanthus nasutus* (L.) Kurz	Shrub	Leaves<br>Leaves, Stem	Decoction<br>Decoction	Oral ingestion<br>Oral ingestion	Single used<br>Single used	3	2	0.0208	LPN<br>CMI	Tai Yong (Sumridpiem, 2017)<br>Tai Yuan (Brun and Schmacher, 1994)
11) *Strobilanthes cusia* (Nees) Kuntze	Shrub	Leaves<br>Non-aerial parts	Decoction<br>Decoction	Oral ingestion<br>Herbal steam	Recipes<br>Recipes	3	3	0.0312	CMI<br>CMI	Karen (Winijchaiyanan, 1995)<br>P. Sumridpiem 2022 4/Tai Yai (N=2)
12) *Thunbergia coccinea* Wall. ex D.Don	Climber	Stem	Decoction	Oral ingestion	Single used	1	1	0.0104	TAK	Karen (Kantasrila, 2016)
13) *Thunbergia laurifolia* L. [a]	Climber	Stem<br>Stem<br>Stem<br>Non-aerial parts<br>Leaves, Stem<br>Leaves<br><br>Leaves<br>Leaves, Stem	Decoction<br>Decoction<br>Decoction<br>Decoction<br>Decoction<br>Decoction,<br>Soaking<br>Decoction<br>Decoction	Oral ingestion<br>Oral ingestion<br>Oral ingestion<br>Oral ingestion<br>Oral ingestion<br>Oral ingestion<br><br>Oral ingestion<br>Herbal steam	Single used<br>Single used<br>Single used<br>Single used<br>Single used<br>Single used<br><br>Recipes<br>Recipes	12	8	0.0833	CMI<br>NAN<br>NAN<br>LPN<br>TAK<br>LPN<br><br>LPN<br>CMI	Tai Yai (Archam, 2011)<br>Khamu (Srithi, 2012)<br>Hmong (Srithi, 2012)<br>Tai Yuan (Inta et al., 2013B)<br>Karen (Kantasrila, 2016)<br>Tai Yuan (Sumridpiem, 2017)<br><br>Tai Yuan (Inta et al., 2020)<br>P. Sumridpiem 2022 84/Tai Yai (N=1)
14) *Sanchezia oblonga* Ruiz & Pav	Shrub*	Leaves	Cooking	Oral ingestion	Food	1	1	0.0104	CMI	P. Sumridpiem 2022 45/Hmong (N=1)
**Acoraceae**										
15) *Acorus calamus* L. [a]	Herb	-	-	-	Recipes	1	1	0.0104	LANNA	Tai Yuan (Manosroi et al., 2013)

Scientific Name	Habit	Part	Preparation	Application	Type of medicine	Use reports	FC	RFC	Study area (Province)	Ethnic (References)/ Voulcher number
16) *Acorus gramineus* Aiton	Herb	Entire plant, Leaves	Decoction, Burning, Cooking	Oral ingestion, Compress, Oral ingestion	Single used, Food	8	5	0.052	CMI	P. Sumridpiem 2023 8/Hmong (N=5)
**Amaranthaceae**										
17) *Iresine diffusa* f. herbstii (Hook.) Pedersen	Herb*	Leaves	Cooking	Oral ingestion	Food	1	1	0.0104	CMI	P. Sumridpiem 2023 10/Hmong (N=1)
18) *Alternanthera bettzickiana* (Regel) G.Nicholson	Herb*	Leaves, Shoots	Cooking	Oral ingestion	Food	2	1	0.0104	CMI	P. Sumridpiem 2023 11/Hmong (N=1)
19) *Achyranthes bidentata* Blume	Herb	Unspecified aerial parts	Cooking	Oral ingestion	Food	1	1	0.0104	CMI	P. Sumridpiem 2022 16/Hmong (N=1)
**Amaryllidaceae**										
20) *Allium cepa* L.[a]	Herb*	Bulb	Powdering	Inhalation or spray	Recipes	2	1	0.0104	LANNA	Tai Yuan (Manosroi et al., 1994)*
21) *Allium sativum* L.[a]	Herb*	Leaves, Bulb<br>Bulb<br>-<br>Bulb	Fresh<br>Fresh<br>-<br>Burning, Pound	Oral ingestion<br>Oral ingestion<br>-<br>Oral ingestion, Poultices	Food<br>Single used<br>Recipes<br>Food, Single used	8	5	0.052	CMI<br>LPN<br>LANNA<br>CMI	Karen (Kaewsangsai, 2017)<br>Tai Yuan (Inta et al., 2013B)<br>Tai Yuan (Manosroi et al., 2013)<br>P. Sumridpiem/Karen (N=2), Hmong (N=2)
22) *Allium tuberosum* Rottler ex Spreng.	Herb*	Leaves	Cooking	Oral ingestion	Food	1	1	0.0104	LPN	Tai Yong (Sumridpiem, 2017)
**Anacardiaceae**										
23) *Mangifera quadrifida* Jack	Tree	Bark	Decoction	Oral ingestion	Recipes	1	1	0.0104	CMI	Karen (Winijchaiyanan, 1995)
24) *Spondias mombin* L.	Tree*	-	-	-	Recipes	1	1	0.0104	LANNA	Tai Yuan (Manosroi et al., 2013)
**Annonaceae**										
25) *Annona muricata* L.[a]	Tree*	Leaves	-	-	-	2	2	0.0208	MSN	Tai Yuan (Inta et al., 2020)<br>Karen (Inta et al., 2020)
**Apiaceae**										
26) *Anethum graveolens* L.	Herb*	Infructescences	Powdering	Inhalation or spray	Recipes	1	1	0.0104	LANNA	Tai Yuan (Manosroi et al., 1994)*
27) *Angelica dahurica* (Hoffm.) Benth. & Hook.f. ex Franch. & Sav. var. dahurica	Herb*	Roots	Decoction	Oral ingestion	Single used	2	2	0.0208	CMI	P. Sumridpiem 2022 34 /Hmong (N=2)
28) *Centella asiatica* (L.) Urb.[a]	Herb	Non-aerial parts, Entire plant<br>Leaves<br>Entire plant<br>Leaves<br>Leaves<br>Non-aerial parts<br>Entire plant	Pounding<br>Decoction<br>Fresh<br>Pounding (mixed with sugar and water)<br>Fresh<br>Decoction, Pounding, Soaking<br>Fresh<br>Infusion (honey), Fresh	Oral ingestion<br>Oral ingestion<br>Oral ingestion<br>Oral ingestion<br>Oral ingestion<br>Oral ingestion<br>Oral ingestion<br>Oral ingestion, Oral ingestion	Single used<br>Recipes<br>Single used<br>Single used<br>Food<br>Single used<br>Food<br>Recipes, Food	11	9	0.0937	CMI<br>CMI<br>CMI<br>TAK<br>TAK<br>LPN<br>CMI<br>CMI	Hmong (Siewphukhieo, 1987)<br>Karen (Winijchaiyanan, 1995)<br>Lisu (Panta, 2015)<br>Karen (Kantasrila, 2016)<br>Karen (Kantasrila, 2016)<br>Tai Yuan (Sumridpiem, 2017)<br>Karen (Panyadee et al., 2019)<br>P. Sumridpiem 2022 14/Karen (N=1),Tai Yai (N=1)
29) *Conioselinum anthriscoides* (H.Boissieu) Pimenov & Kljuykov[a]	Herb*	Roots	Powdering	Oral ingestion	Recipes	2	2	0.0208	LANNA<br>CMI	Tai Yuan (Manosroi et al., 2013)<br>P. Sumridpiem 2022 92/ TY(N=1)
30) *Coriandrum sativum* L.[a]	Herb*	-	-	-	Recipes	1	1	0.0104	LANNA	Tai Yuan (Manosroi et al., 2013)

Scientific Name	Habit	Part	Preparation	Application	Type of medicine	Use reports	FC	RFC	Study area (Province)	Ethnic (References)/ Voucher number
31) *Cuminum cyminum* L.	Herb*	Infructescences	Powdering	Inhalation or spray	Recipes	2	1	0.0104	LANNA	Tai Yuan (M anosroi et al., 1994)*
32) *Foeniculum vulgare* Mill. subsp. *vulgare* var. *dulce* (Mill.) Thell.	Herb*	Infructescences	Powdering	Inhalation or spray	Recipes	2	1	0.0104	LANNA	Tai Yuan (Manosroi et al., 1994)*
33) Ligusticum sinense Oliv. cv. Chuanxiong*	Herb*	Roots	Decoction	Oral ingestion	Single used	2	2	0.0208	CMI	P. Sumridpiem 2022 12 /Hmong (N=2)
34) *Oenanthe javanica* (Blume) DC.	Herb	Leaves	Decoction	Oral ingestion	Single used	1	1	0.0104	NAN	Tai Yuan (Songsangchun, 2015)
35) *Seseli mairei* H.Wolff**	Herb	Underground	-	-	-	1	1	0.0104	CRI	Akha (Srisanga et al., 2011)
**Apocynaceae**										
36) *Asclepias curassavica* L.	Herb*	-	-	-	Recipes	1	1	0.0104	LANNA	Tai Yuan (Manosroi et al., 2013)
37) *Amphineurion marginatum* (Roxb.) D.J.Middleton	Climber	Non-aerial parts	Decoction	Oral ingestion, Herbal steam, Bath	Recipes	6	2	0.0208	CMI	P. Sumridpiem 2022 25/ Tai Yai (N=2)
38) *Tabernaemontana pandacaqui* Poir.	Tree	Stem	Decoction	Oral ingestion	Single used	1	1	0.0104	NAN	Hmong (Srithi, 2012)
39) *Gymnema inodorum* (Lour.) Decne.	Climber	Leaves<br><br>Leaves	Decoction<br>Cooking,<br>Pill, Pounding	Oral ingestion<br><br>Oral ingestion	Recipes<br><br>Food,<br>Single used	4	2	0.0208	LPN<br><br>LPN	Tai Yuan (Inta et al., 2020)<br><br>Tai Yuan (Sumridpiem, 2017)
40) *Rauvolfia verticillata* (Lour.) Baill.	Shrub	Roots	-	-	-	1	1	0.0104	LANNA	Tai Yuan (Manosroi et al., 2009)
**Araceae**										
41) *Pothos chinensis* (Raf.) Merr.	Climber	Entire plant	Decoction	Oral ingestion	Single used	1	1	0.0104	CMI	P. Sumridpiem 2022 70 / Karen (N=1)
**Araliaceae**										
42) *Hydrocotyle javanica* Thunb.	Herb	Arial stem<br>Entire plant	Fresh<br>Fresh	Oral ingestion<br>Oral ingestion	Single used<br>Single used	2	2	0.0208	CMI<br>TAK	Hmong (Nguanchoo, 2014)<br>Karen (Kantasrila, 2016)
43) *Trevesia palmata* (Roxb. ex Lindl.) Vis.	Tree	Leaves	Burning	Poultices	Single used	1	1	0.0104	CMI	P. Sumridpiem /Hmong (N=1)
**Arecaceae**										
44) *Borassus flabellifer* L.	Tree	Leaves	Powdering	Inhalation or spray	Recipes	2	1	0.0104	LANNA	Tai Yuan (Manosroi et al., 1994)*
**Asparagaceae**										
45) *Dracaena cochinchinensis* (Lour.) S.C.Chen	Shrub	-	-	-	Recipes	1	1	0.0104	LANNA	Tai Yuan (Manosroi et al., 2013)
**Asteraceae**										
46) *Ageratina adenophora* (Spreng.) R.M.King & H.Rob.	Herb*	Shoots	Pounding	Liniment	Recipes	1	1	0.0104	CMI	P. Sumridpiem /Hmong (N=1)
47) *Artemisia lactiflora* Wall. ex DC.	Herb*	Shoots, Leaves, Inflorescences	Cooking	Oral ingestion	Food	7	4	0.0416	CMI	P. Sumridpiem 2022 40/Hmong (N=4)
48) *Artemisia roxburghiana* Besser	Herb	Non-aerial parts	Soaking	Wiping the body	Single used	1	1	0.0104	CMI	P. Sumridpiem 2022 24 / Lisu (N=1)
49) *Artemisia vulgaris* L.	Herb*	Shoots, Leaves	Pounding	Poultices	Recipes	2	1	0.0104	CMI	P. Sumridpiem 2022 42 /Hmong (N=1)

Scientific Name	Habit	Part	Preparation	Application	Type of medicine	Use reports	FC	RFC	Study area (Province)	Ethnic (References)/Voucher number
50) *Blumea balsamifera* (L.) DC.[a]	Shrub	Leaves<br>Leaves, Shoots	-<br>Burning, Decoction, Soaking, Pounding	-<br>Oral ingestion, Herbal steam, Bath (body, head, and face), Wiping the body, Poultices, Liniment	Recipes<br>Recipes, Single used	27	19	0.1979	LANNA<br>CMI	Tai Yuan (Manosroi et al., 2013)<br>P. Sumridpiem 2022 23 /Tai Yai (N=6),Karen (N=6), Lisu (N=1), Hmong(N=5)
51) *Chromolaena odorata* (L.) R.M.King & H.Rob.[a]	Herb*	Roots<br>Leaves<br>Leaves	Decoction<br>Fresh<br>Fresh	Oral ingestion<br>Oral ingestion<br>Oral ingestion	Single used	3	3	0.0312	CMI<br>MSN<br>MSN	Tai Yuan (Brun and Schmacher, 1994)<br>Lua (Inta et al., 2020)<br>Tai Yuan (Inta et al., 2020)
52) *Cyanthillium cinereum* (L.) H.Rob.	Herb	Entire plant<br>Entire plant	Decoction<br>Decoction	Oral ingestion<br>Oral ingestion	Single used<br>Single used	2	2	0.0208	LPN<br>NAN	Tai Yuan (Sumridpiem, 2017)<br>Tai Lue (Tovaranonte, 1998)
53) *Dichrocephala integrifolia* (L.f.) Kuntze	Herb	Entire plant	Decoction	Herbal steam	Recipes	1	1	0.0104	CMI	P. Sumridpiem 2022 38 / Tai Yai (N=1)
54) *Dolomiaea costus* (Falc.) Kasana & A.K.Pandey[a]	Herb*	Roots	Powdering	Oral ingestion	Recipes	3	3	0.0312	CMI	P. Sumridpiem 2022 20 / Tai Yai (N=3)
55) *Eclipta prostrata* (L.) L.	Herb	Entire plant	Decoction	Herbal steam	Recipes	1	1	0.0104	CMI	P. Sumridpiem 2022 102 / Tai Yai (N=1)
56) *Gymnanthemum extensum* (DC.) Steetz	Shrub	Entire plant<br>Bark<br>Leaves<br>Entire plant, Stem, Leaves	Decoction<br>Decoction<br>Fresh<br>Decoction	Oral ingestion<br>Oral ingestion<br>Oral ingestion<br>Herbal steam, Oral ingestion, Bath	Single used<br>Single used<br>Single used<br>Recipes	8	5	0.052	PYO<br>CMI<br>CRI<br>CMI	Tai Yuan (Inta et al., 2019)<br>Karen (Biodiversity-based economy development office, 2022)<br>Lisu (Biodiversity-based economy development office, 2022)<br>P. Sumridpiem 2022 19 / Tai Yai (N=2), Lisu (N=1)
57) *Gynura bicolor* (Roxb. ex Willd.) DC.	Herb	Leaves, Shoots	Cooking	Oral ingestion	Food	2	1	0.0104	CMI	P. Sumridpiem 2023 36 /Hmong (N=1)
58) *Gynura nepalensis* DC.	Herb	Leaves	Decoction	Oral ingestion	Food	1	1	0.0104	NAN	Hmong (Srithi, 2012)
59) *Gynura procumbens* Merr.	Climber	Leaves<br>Leaves	Fresh<br>Fresh	Oral ingestion<br>Oral ingestion	Food<br>Food	2	2	0.0208	NAN	Khamu (Srithi, 2012)<br>Lua (Srithi, 2012)
60) *Helianthus annuus* L.	Herb*	Leaves, Inflorescences	Decoction	Bath (head and face)	Single used	2	1	0.0104	CMI	P. Sumridpiem /Hmong (N=1)
61) *Laggera crispata* (Vahl) Hepper & J.R.I.Wood	Herb	Leaves, Shoots	Burning	Compress, Poultices	Single used	5	3	0.0312	CMI	P. Sumridpiem 2022 54/Hmong (N=3)
62) *Sphagneticola trilobata* (L.) Pruski	Climber	Entire plant	Decoction	Herbal steam	Recipes	1	1	0.0104	CMI	P. Sumridpiem/ Tai Yai (N=1)
**Basellaceae**										
63) *Anredera cordifolia* (Ten.) Steenis	Herb	Leaves, Shoots<br>Leaves, Shoots	Fresh<br>Cooking	Oral ingestion<br>Oral ingestion	Single used<br>Food	4	2	0.0208	CMI<br>CMI	Hmong (Nguanchoo, 2014)<br>P. Sumridpiem 2023 /Hmong (N=1)
64) *Basella alba* L.	Climber	Inflorescences, Shoots<br>Leaves, Shoots	Cooking<br>Cooking	Oral ingestion<br>Oral ingestion	Food<br>Food	4	2	0.0208	LPN<br>CMI	Tai Yong (Sumridpiem, 2017)<br>P. Sumridpiem/Hmong (N=1)
**Betulaceae**										
65) *Betula alnoides* Buch.-Ham. ex D.Don	Tree	Leaves<br>Leaves	Fresh<br>Fresh	Oral ingestion<br>Oral ingestion	Single used<br>Single used	2	2	0.0208	LPN<br>LPN	Tai Yuan (Inta et al., 2020)<br>Karen (Inta et al., 2020)
**Bignoniaceae**										
66) *Mayodendron igneum* (Kirz) Kirz	Tree	Bark	Decoction	Oral ingestion	Single used	1	1	0.0104	MSN	Karen (Inta et al., 2018C)

Scientific Name	Habit	Part	Preparation	Application	Type of medicine	Use reports	FC	RFC	Study area (Province)	Ethnic (References)/Voucher number
67) *Millingtonia hortensis* L.f.	Tree	Leaves	Decoction	Oral ingestion, Herbal steam, Bath	Recipes	6	2	0.0208	CMI	P. Sumridpiem/ Tai Yai (N=2)
68) *Oroxylum indicum* (L.) Kurz [a]	Tree	Bark	Decoction	Oral ingestion	Single used	1	1	0.0104	CMI	Karen (Tangjitman., 2014)
**Boraginaceae**										
69) *Heliotropium indicum* L.	Herb	Roots	Decoction	Oral ingestion	Single used	1	1	0.0104	MSN	Karen (Inta et al., 2018A)
**Brassicaceae**										
70) *Brassica juncea* (L.) Czern	Herb*	Leaves	Burning	Poultices	Single used	1	1	0.0104	CMI	P. Sumridpiem/ Hmong (N=1)
71) *Lepidium sativum* L.	Herb*	Infructescences	Powdering	Inhalation or spray	Recipes	2	1	0.0104	LANNA	Tai Yuan (Manosroi et al., 1994)*
**Bromeliaceae**										
72) *Ananas comosus* (L.) Merr.	Herb*	Infructescences	Fresh	Oral ingestion	Single used	1	1	0.0104	LPN	Tai Yuan (Sumridpiem, 2017)
**Calophyllaceae**										
73) *Mesua ferrea* L.[a]	Tree	-	-	-	Recipes	1	1	0.0104	LANNA	Tai Yuan (Manosroi et al., 2013)
**Campanulaceae**										
74) *Lobelia angulata* G. Forst.	Herb*	Entire plant	Fresh, Decoction	Oral ingestion	Single used	2	1	0.0104	CMI	Hmong (Nguanchoo, 2014)
**Capparaceae**										
75) *Capparis micracantha* DC.[a]	Shrub	-	-	-	Recipes	1	1	0.0104	LANNA	Tai Yuan (Manosroi et al., 2013)
76) *Crateva magna* (Lour.) DC.	Tree	Leaves	Mix with cow dung	Poultice	Recipes	1	1	0.0104	AKHA	Akha (Biodiversity-based economy development office, 2022)
77) *Crateva adansonii* DC. subsp. *trifoliata* (Roxb.) Jacobs/	Tree	Bark	Decoction	Herbal steam	Recipes	1	1	0.0104	CMI	P. Sumridpiem 2022 32 / Tai Yai (N=1)
**Cardiopteridaceae**										
78) *Gonocaryum lobbianum* (Miers) Kurz	Shrub	Infructescences	Burned	Oral ingestion	Food	1	1	0.0104	NAN	Mien (Srithi, 2012)
**Caricaceae**										
79) *Carica papaya* L.	Tree*	Infructescences (green fruit without peel)<br>Roots	Decoction<br>Powdering	Oral ingestion<br>Oral ingestion	Single used<br>Recipes	2	2	0.0208	LPN<br>CMI	Tai Yuan (Sumridpiem, 2017)<br>P. Sumridpiem/ Tai Yai (N=1)
**Combretaceae**										
80) *Combretum latifolium* Blume	Climb er	-	-	-	Recipes	1	1	0.0104	LANNA	Tai Yuan (Manosroi et al., 2013)
81) *Terminalia chebula* Retz.[a]	Tree	-	-	-	Recipes	1	1	0.0104	LANNA	Tai Yuan (Manosroi et al., 2013)
**Commelinaceae**										
82) *Murdannia edulis* (Stokes) Faden	Herb	Entire plant	Decoction	Oral ingestion	Single used	1	1	0.0104	MSN	Tai Yai (Phongloy, 2015)
83) *Murdannia loriformis* (Hassk.) R.S.Rao & Kammathy	Herb	Leaves (fresh)	Crushed	Oral ingestion	Recipes	1	1	0.0104	LPN	Tai Yuan (Sumridpiem, 2017)
84) *Rourea minor* (Gaertn.) Merr.	Climb er	Stem, Leaves, Roots	Decoction, Infusion	Oral ingestion Herbal steam, Bath	Recipes	8	2	0.0208	CMI	P. Sumridpiem 2022 7/ Lisu (N=2)
85) *Tradescantia spathaceae* Sw.	Herb*	Leaves (fresh)	Crushed	Oral ingestion	Recipes	1	1	0.0104	LPN	Tai Yuan (Sumridpiem, 2017)
86) *Tradescantia zebrina* Bosse	Herb*	Leaves, Shoots	Cooking	Oral ingestion	Food	2	1	0.0104	CMI	P. Sumridpiem 2022 17/Hmong (N=1)
87) *Kalanchoe blossfeldiana* Poelln.	Herb*	Leaves	Cooking	Oral ingestion	Food	1	1	0.0104	CMI	P. Sumridpiem/Hmong (N=1)

Scientific Name	Habit	Part	Preparation	Application	Type of medicine	Use reports	FC	RFC	Study area (Province)	Ethnic (References)/Voucher number
**Cucurbitaceae**										
88) *Gynostemma pentaphyllum* (Thunb.) Makino	Climber	Leaves,<br>Non-aerial parts<br>Entire plant<br>Leaves<br>Entire plant	Decoction<br><br>Decoction<br>Soaking<br>Decoction	Oral ingestion<br><br>Oral ingestion<br>Oral ingestion<br>Oral ingestion	Single used<br><br>Single used<br>Single used<br>Single used	5	4	0.0416	NAN<br><br>LPN<br>PYO<br>MSN	Hmong (Srithi, 2012)<br><br>Yuan (Inta et al., 2019)<br>Yuan (Inta et al., 2020)<br>Karen (Inta et al., 2018B)
89) *Momordica charantia* L. [a]	Climber	Infructescences<br>Leaves	Fresh<br>Fresh	Oral ingestion<br>Oral ingestion	Single used<br>Single used	2	2	0.0208	LPN<br>MSN	Tai Yuan (Inta et al., 2020)<br>Tai Yuan (Inta et al., 2020)
90) *Momordica cochinchinensis* (Lour.) Spreng.[a]	Climber	Infructescences	Decoction	Oral ingestion	Single used	1	1	0.0104	LPN	Tai Yuan (Sumridpiem, 2017)
**Cyperaceae**										
91) *Cyperus rotundus* L. [a]	Herb	-	-	-	Recipes	1	1	0.0104	LANNA	Tai Yuan (Manosroi et al., 2013)
**Dioscoreaceae**										
92) *Tacca chantrieri* André [a]	Herb	Underground parts, Leaves	Infusion	Oral ingestion	Single used	2	1	0.0104	PYO	Tai Yuan (Inta et al., 2019)
**Ebenaceae**										
93) *Diospyros decandra* Lour. [a]	Tree	-	-	-	Recipes	1	1	0.0104	LANNA	Tai Yuan (Manosroi et al., 2013)
**Euphorbiaceae**										
94) *Codiaeum variegatum* (L.) Rumph. ex A.Juss.	Shrub*	Leaves	Decoction	Oral ingestion	Single used	1	1	0.0104	MSN	Tai Yuan (Inta et al., 2020)
95) *Croton crassifolius* Geiseler	Shrub	-	-	-	Recipes	1	1	0.0104	LANNA	Tai Yuan (Manosroi et al., 2013)
96) *Plukenetia volubilis* L.	Climber	Infructescences	Decoction	Oral ingestion	Recipes	1	1	0.0104	CMI	P. Sumridpiem 2022 74/ Lisu (N=1)
97) *Ricinus communis* L.	Shrub*	Leaves, Shoots, Stem	Burning, Decoction	Blow smoke into ears, Oral ingestion, Poultices	Single used	7	3	0.0312	CMI	P. Sumridpiem 2022 8 /Karen (N=1), Hmong (N=2)
**Fabaceae**										
98) *Adenanthera pavonina* L.	Tree	Leaves	Decoction	Oral ingestion	Single used	1	1	0.0104	CMI	Karen (Sutjaritjai, 2019)
99) *Albizia myriophylla* Benth	Tree	Stems	Decoction<br>Powdered	Oral ingestion	Recipes	2	2	0.0208	CMI	P. Sumridpiem 2022 17 / Tai Yai (N=2)
100) *Biancaea sappan* (L.) Tod. [a]	Tree	Stems<br>Infructescence, Peduncle, Seeds, Stems	Decoction<br>Decoction	Oral ingestion<br>Oral ingestion, Powdered	Single used<br>Single used	9	6	0.0625	NAN<br>CMI	Mien (Srithi, 2012)<br>P. Sumridpiem 2022 16 /Karen (N=2), Hmong(N=3)
101) *Butea superba* Roxb. ex Willd.	Climber	Stems	Decoction	Herbal steam, Bath	Recipes	2	1	0.0104	CMI	P. Sumridpiem 2022 27 / Tai Yai (N=1)
102) *Caesalpinia pulcherrima* (L.) Sw.	Shrub*	Seed	Fresh	Oral ingestion	Single used	1	1	0.0104	LPN	Tai Yuan (Sumridpiem, 2017)
103) *Campylotropis parviflora* (Kurz) Schindl.	Shrub	Roots	Decoction	Oral ingestion	Single used	1	1	0.0104	CMI	Hmong (Highland Research, 2018)
104) *Canavalia gladiata* (Jacq.) DC.	Climber	Infructescences	Decoction	Oral ingestion	Single used	1	1	0.0104	CMI	Hmong (Nguanchoo, 2014)
105) *Cassia fistula* L. [a]	Tree	Leaves<br>-<br>Roots	Decoction<br>-<br>Powdering	Oral ingestion<br>-<br>Oral ingestion	Single used<br>Recipes<br>Recipes	3	3	0.0312	CMI<br>LANNA<br>CMI	Karen (Sutjaritjai, 2019)<br>Tai Yuan (Manosroi et al., 2013)<br>P. Sumridpiem 2023 14 / Tai Yai (N=1)
106) *Clitoria ternatea* L.	Climber*	Inflorescences	Decoction	Oral ingestion	Single used	2	2	0.0208	LPN	Tai Yong (Sumridpiem, 2017)<br>Tai Yuan (Sumridpiem, 2017)

Scientific Name	Habit	Part	Preparation	Application	Type of medicine	Use reports	FC	RFC	Study area (Province)	Ethnic (References)/Voucher number
107) *Glycyrrhiza glabra* L.	Shrub*	-	-	-	Recipes	1	1	0.0104	LANNA	Tai Yuan (Manosroi et al., 2013)
108) *Lablab purpureus* (L.) Sweet	Climber	Entire plant	Decoction	Oral ingestion	Single used	1	1	0.0104	CMI	Karen (Sutjaritjai, 2019)
109) *Lysiphyllum strychnifolium* (Craib) A.Schmitz	Climber	Leaves	Decoction	Oral ingestion	Recipes	1	1	0.0104	LPN	Tai Yuan (Inta et al., 2020)
110) *Mimosa diplotricha* var. diplotricha	Herb*	Entire plant<br>Leaves, Stem	Decoction<br>Decoction	Oral ingestion<br>Oral ingestion	Single used<br>Single used	3	2	0.0208	NAN<br>NAN	Mien (Tovaranonte., 1998)<br>Mien (Panyaphu., 2012)
111) *Mimosa pudica* L.	Shrub*	Entire plant<br>Entire plant<br>Entire plant<br>Entire plant<br>Entire plant<br>Entire plant<br>Entire plant<br>Entire plant	Decoction<br>Decoction<br>Decoction<br>Decoction<br>Decoction<br>Decoction<br>Decoction<br>Decoction	Oral ingestion<br>Oral ingestion<br>Oral ingestion<br>Oral ingestion<br>Oral ingestion<br>Oral ingestion<br>Oral ingestion<br>Oral ingestion, Herbal steam, Bath	Single used<br>Single used<br>Single used<br>Single used<br>Single used<br>Single used<br>Single used<br>Recipes	12	12	0.125	NAN<br>NAN<br>LPN<br>PRE<br>CMI<br>MSN<br>LPN<br>CMI	Mien (Srithi, 2012)<br>Khamu (Srithi, 2012)<br>Tai Yuan (Sumridpiem, 2017)<br>Tai Yuan (Inta et al., 2019)<br>Karen (Sutjaritjai, 2019)<br>Tai Yuan (Inta et al., 2020)<br>Tai Yuan (Inta et al., 2020)<br>P. Sumridpiem 2023 18 / Tai Yai (N=3), Karen (N=2)
112) *Neustanthus phaseoloides* (Roxb.)	Climber	Roots	Decoction	Oral ingestion	Single used	1	1	0.0104	CMI	Karen (Sutjaritjai, 2019)
113) *Ototropis multiflora* (DC.) H. Ohashi & K.Ohashi/	Shrub	Roots	Decoction	Oral ingestion	Oral ingestion	1	1	0.0104	CMI	P. Sumridpiem 2022 65 /Lisu (N=1)
114) *Phanera ornata* (Kurz) Thoth. var. *kerrii* (Gagnep.) Bandyop., P.P. Ghoshal & M.K. Pathak	Climber	Roots	Decoction	Oral ingestion	Single used	1	1	0.0104	CMI	Karen (Sutjaritjai, 2019)
115) *Pithecellobium dulce* (Roxb.) Benth.	Tree*	Roots (fresh)<br>Roots (fresh)	Crushing<br>Crushing	Oral ingestion<br>Oral ingestion	Single used<br>Single used	2	2	0.0208	CMI<br>LPN	Tai Yuan (Brun and Schmacher, 1994)<br>Tai Yuan (Brun and Schmacher, 1994)
116) *Polhillides velutina* (Willd.) H. Ohashi & K. Ohashi	Shrub	Stem<br>Leaves	Decoction<br>Decoction	Oral ingestion<br>Oral ingestion	Single used<br>Single used	2	2	0.0208	CMI<br>MSN	Tai Yai (Phongloy, 2015)<br>Tai Yai (Phongloy, 2015)
117) *Pterocarpus santalinus* L.f.	Tree*	Stem	Powdering	Oral ingestion	Recipes	1	1	0.0104	CMI	P. Sumridpiem 2022 82 / Tai Yai (N=1)
118) *Senegalia pennata* (L.) Maslin	Climber	Bark	Decoction	Herbal steam	Recipes	1	1	0.0104	CMI	P. Sumridpiem/ Tai Yai (N=1)
119) *Senna siamea* (Lam.) H.S. Irwin & Barneby [a]	Tree	Leaves, Inflorescences<br>Leaves, Inflorescences<br>-	Decoction<br>Cooking<br>-	Oral ingestion<br>Oral ingestion<br>-	Single used<br>Single used<br>Recipes	5	4	0.0416	CMI, LPN<br>CMI<br>LANNA	Tai Yuan (Inta et al., 2013B)<br>Karen (Sutjaritjai, 2019)<br>Tai Yuan (Manosroi et al., 2013)
120) *Uraria oblonga* (Wall. ex Benth.) H. Ohashi & K. Ohashi	Shrub	Entire plant<br>Roots	Decoction<br>Decoction	Oral ingestion<br>Bath	Recipes<br>Recipes	2	2	0.0208	CMI<br>CMI	Tai Lue (Muangyen, 2013)<br>P.Sumridpiem 2022 73 / Tai Yai (N=1)
**Hypoxidaceae**										
121) *Curculigo gracilis* (Kurz) Wall. ex Hook.f.	Herb	Entire plant	-	-	-	1	1	0.0104	MSN	Tai Yai (Inta et al., 2018D)
122) *Curculigo latifolia* Dryand. ex W.T. Aiton	Herb	Entire plant<br>Entire plant	Decoction<br>Decoction	Bath<br>Bath	Single used<br>Single used	2	2	0.0208	CMI<br>MSN	Tai Yai (Phongloy, 2015)<br>Tai Yai (Phongloy, 2015)
**Lamiaceae**										
123) *Agastache rugosa* (Fisch. & C.A. Mey.) Kuntze	Herb*	Leaves, Shoots	Decoction, Pounding	Liniment (temporal area)	Recipes, Single used	3	2	0.0208	CMI	P. Sumridpiem 2022/Hmong (N=2)
124) *Clerodendrum chinense* (Osbeck) Mabb.	Shrub	Roots	Decoction	Oral ingestion	Recipes	7	5	0.052	CMI	P. Sumridpiem 2022 5 /Lisu (N=5)
125) *Clerodendrum glandulosum* Lindl.	Shrub	Leaves, Shoots	Burning, Decoction	Poultices, Herbal steam	Recipes, Single used	5	3	0.0312	CMI	P. Sumridpiem 2022 29 /Lisu (N=1), ,Hmong (N=2)

Scientific Name	Habit	Part	Preparation	Application	Type of medicine	Use reports	FC	RFC	Study area (Province)	Ethnic (References)/Voucher number
126) *Clerodendrum indicum* (L.) Kuntze. [a]	Shrub	Roots	Decoction, Powdering	Oral ingestion	Single used	2	1	0.0104	LPN	Tai Yai (Inta et al., 2020)
127) *Clerodendrum paniculatum* L.	Shrub	Roots	Powdering	Oral ingestion	Recipes	1	1	0.0104	CMI	P. Sumridpiem 2022 91 / Tai Yai (N=1)
128) *Coleus scutellarioides* (L.) Benth.	Herb*	Leaves	Dry and Decoction	Oral ingestion	Single used	1	1	0.0104	CRI	Tai Yuan (Tovaranonte, 2002)
129) *Elsholtzia penduliflora* W.W.Sm.	Herb	Leaves	Cooking	Oral ingestion	Food	3	3	0.0312	CMI	P. Sumridpiem 2023 /Hmong (N=3)
130) *Mentha × piperita* L.	Herb*	Leaves	Decoction	Oral ingestion	Single used	1	1	0.0104	CMI	Hmong (Biodiversity-based economy development office, 2022)
131) *Mentha × villosa* Huds.	Herb*	Entire plant	Fresh	Oral ingestion	Single used	1	1	0.0104	CMI	Karen (Winijchaiyanan., 1995)
132) *Orthosiphon aristatus* (Blume) Miq.[a]	Herb	Entire plant<br>Leaves, Shoots	Decoction<br>Decoction	Oral ingestion<br>Oral ingestion	Recipes<br>Recipes	3	2	0.0208	CMI<br>LPN	Tai Lue (Muangyen, 2013)<br>Tai Yuan (Sumridpiem, 2017)
133) *Rotheca serrata* (L.) Steane & Mabb.	Shrub	Leaves, Roots	Honey infusion	Oral ingestion, Herbal steam, Bath	Recipes	11	5	0.052	CMI	P. Sumridpiem 2022 30 / Tai Yai (N=3), LS (N=1), Karen (N=1)
134) *Vitex trifolia* L. [a]	Shrub	-<br>Leaves	-<br>Decoction	-<br>Herbal steam	Recipes<br>Recipes	2	2	0.0208	LANNA<br>CMI	Tai Yuan (Manosroi et al., 2013)<br>P. Sumridpiem 2022 90 /Karen (N=1)
**Lauraceae**										
135) *Actinodaphne henryi* Gamble	Tree	Leaves	Decoction	Herbal steam	Recipes	1	1	0.0104	CMI	P. Sumridpiem 2022 54 /Karen (N=1)
136) *Litsea cubeba* (Lour.) Pers	Tree	Stem	Decoction	Oral ingestion	Recipes	1	1	0.0104	CMI	P. Sumridpiem 2022 44 /Karen (N=1)
**Lecythidaceae**										
137) *Careya arborea* Roxb.	Tree	Leaves	Decoction	Bath	Single used	1	1	0.0104	MSN	Tai Yai (Phongloy, 2015)
**Loranthaceae**										
138) *Dendrophthoe pentandra* (L.) Miq. [a]	Shrub	Entire plant<br>Stem	Decoction<br>-	Oral ingestion<br>-	Recipes<br>Recipes	2	2	0.0208	CMI<br>NAN	Tai Lue (Muangyen, 2013)<br>Tai Yuan (Inta et al., 2014)
**Lythraceae**										
139) *Lawsonia inermis* L.	Shrub*	-	-	-	Recipes	1	1	0.0104	LANNA	Tai Yuan (Manosroi et al., 2013)
**Malvaceae**										
140) *Abutilon indicum* (L.) Sweet	Shrub	Leaves<br>Infructescences	Decoction<br>Decoction	Oral ingestion<br>Oral ingestion	Single used<br>Single used	2	2	0.0208	CMI<br>LPN	Tai Yuan (Pomnat, 2013)<br>Tai Yuan (Inta et al.,2013B)
141) *Helicteres isora* L.	Shrub	Infructescences	Decoction	Oral ingestion	Single used	2	1	0.0104	PYO, PRE	Tai Yuan (Inta et al., 2019)
**Marantaceae**										
142) *Maranta arundinacea* L.	Herb*	Rhizomes	Decoction	Oral ingestion	Single used	1	1	0.0104	CMI	Karen (Sukkho, 2008)
**Marsileaceae**										
143) *Marsilea minuta* var. *minuta*	Aquatic fern	Entire plant	Fresh	Oral ingestion	Single used	1	1	0.0104	CMI	P. Sumridpiem 2022 45 /Karen (N=1)
**Melastomataceae**										
144) *Melastoma malabathricum* subsp. *normale* (D.Don) Karst.Mey.	Shrub	Roots	Decoction	Oral ingestion	Single used	1	1	0.0104	CRI	Akha (Srisanga et al., 2011)
145) *Osbeckia stellata* Buch.-Ham. ex D.Don	Shrub	Roots	-	-	Single used	1	1	0.0104	CRI	Akha (Srisanga et al., 2011)
**Meliaceae**										
146) *Azadirachta indica* A. Juss. [a]	Tree	Young leaves, Inflorescences	Fresh	Oral ingestion	Single used	2	1	0.0104	PYO	Tai Yuan (Inta et al., 2019)
**Menispermaceae**										
147) *Stephania venosa* (Blume) Spreng.	Climber	Roots	Decoction	Oral ingestion	Recipes	1	1	0.0104	CMI	P. Sumridpiem 2022 97/ Tai Yai (N=1)

Scientific Name	Habit	Part	Preparation	Application	Type of medicine	Use reports	FC	RFC	Study area (Province)	Ethnic (References)/Voucher number
148) *Tiliacora triandra* (Colebr.) Diels [a]	Climber	Leaves (fresh)<br>Leaves (fresh)<br>Leaves<br>Leaves (fresh)<br>Leaves (fresh)<br><br>-	Crushed<br>Crushed<br>Decoction<br>Crushed<br>Crushed with other plants<br>-	Oral ingestion<br>Oral ingestion<br>Oral ingestion<br>Oral ingestion<br>Oral ingestion<br><br>-	Single used<br>Single used<br>Single used<br>Single used<br>Recipes<br><br>Recipes	6	5	0.052	LPN<br>NAN<br>LPN<br>LPN<br>LPN<br><br>LANNA	Tai Yuan (Ethnobotany, 2015)<br>Tai Yuan (Inta et al., 2014)<br>Tai Yong (Sumridpiem, 2017)<br>Tai Yuan (Sumridpiem, 2017)<br>Tai Yuan (Sumridpiem, 2017)<br><br>Tai Yuan (Manosroi et al., 2013)
149) *Tinospora crispa* (L.) Hook.f. & Thomson [a]	Climber	Stems<br>-<br>Stems	Honey infusion<br>-<br>Decoction, Honey infusion	Oral ingestion<br>-<br>Oral ingestion	Single used<br>Recipes<br>Single used, Recipes	4	4	0.0417	LPN<br>LANNA<br>CMI	Tai Yuan (Sumridpiem, 2017)<br>Tai Yuan (Manosroi et al., 2013)<br>P. Sumridpiem 2022 3/ Karen(N=1), Tai Yai (N=1)
**Moraceae**										
150) *Artocarpus heterophyllus* Lam.	Tree*	-	-	-	Recipes	1	1	0.0104	LANNA	Tai Yuan (Manosroi et al., 2013)
151) *Ficus racemosa* L. [a]	Tree	-	-	-	Recipes	1	1	0.0104	LANNA	Tai Yuan (Manosroi et al., 2013)
152) *Ficus simplicissima* Lour.	Shrub*	Roots	Decoction	Oral ingestion	Single used	1	1	0.0104	NAN	Hmong (Srithi, 2012)
153) *Morus alba* L. [a]	Shrub*	Leaves<br>Non-aerial parts, Leaves	Decoction<br>Decoction, Burning	Oral ingestion<br>Bath, Oral ingestion, Poultices	Single used<br>Recipes, Single used	6	6	0.0625	LPN<br>CMI	Tai Yuan (Sumridpiem, 2017)<br>P. Sumridpiem 2023 5/ Tai Yai (N=2), Lisu (N=1), Hmong (N=2)
**Moringaceae**										
154) *Moringa oleifera* Lam. [a]	Tree	Leaves<br>-<br>Leaves, Seeds Infructescences,<br>Leaves<br>Leaves<br>Infructescences<br>Seed	Powdered<br>-<br>Fresh<br><br>Pill<br>Cooking<br>Cooking<br>Drying	Oral ingestion<br>-<br>Oral ingestion<br><br>Oral ingestion<br>Oral ingestion<br>Oral ingestion<br>Oral ingestion	Single used<br>Recipes<br>Single used<br><br>Single used<br>Food<br>Food<br>Single used	9	4	0.0416	NAN<br>LANNA<br>MSN<br><br>LPN<br>LPN<br>LPN<br>LPN	Hmong (Srithi, 2012)<br>Tai Yuan (Manosroi et al., 2013)<br>Tai Yuan (Inta et al., 2020)<br><br>Tai Yuan (Sumridpiem, 2017)<br>Tai Yuan (Sumridpiem, 2017)<br>Tai Yuan (Sumridpiem, 2017)<br>Tai Yuan (Sumridpiem, 2017)
**Musaceae**										
155) *Musa balbisiana* Colla	Herb*	Leaves	Decoction	Herbal steam	Recipes	1	1	0.0104	CMI	P. Sumridpiem 2022 50 /Karen (N=1)
156) *Musa × paradisiaca* L.	Herb	Infructescences	Honey infusion	Oral ingestion	Recipes	1	1	0.0104	CMI	P. Sumridpiem/ Tai Yai (N=1)
**Myrtaceae**										
157) *Syzygium aromaticum* (L.) Merr. & L.M.Perry [a]	Shrub*	-	-	-	Recipes	1	1	0.0104	LANNA	Tai Yuan (Manosroi et al., 2013)
**Nelumbonaceae**										
158) *Nelumbo nucifera* Gaertn.[a]	Herb	Seed (embryo)<br>-	Decoction<br>-	Oral ingestion<br>-	Single used<br>Recipes	2	2	0.0208	LPN<br>LANNA	Tai Yuan (Sumridpiem, 2017)<br>Tai Yuan (Manosroi et al., 2013)
**Oleaceae**										
159) *Jasminum grandiflorum* L.	Climber*	Non-aerial parts	Decoction	Oral ingestion, Herbal steam, Bath	Recipes	6	2	0.0208	CMI	P. Sumridpiem 2022 10 / Tai Yai (N=2)
**Ophioglossaceae**										
160) *Helminthostachys zeylanica* (L.) Hook.	Terrestrial Fern	Young leaves, Roots	Decoction	Oral ingestion	Single used	2	1	0.0104	CMI	Tai Yuan (Inta et al., 2013A)
**Oxalidaceae**										
161) *Averrhoa carambola* L.	Tree*	Infructescences	Fresh	Oral ingestion	Single used	1	1	0.0104	LPN	Tai Yuan (Sumridpiem, 2017)

Scientific Name	Habit	Part	Preparation	Application	Type of medicine	Use reports	FC	RFC	Study area (Province)	Ethnic (References)/Voucher number
**Pandanaceae**										
162) *Pandanus amaryllifolius* Roxb. ex Lindl.[a]	Shrub*	Roots<br>Leaves (fresh)	Decoction<br>Crushed	Oral ingestion<br>Oral ingestion	Single used<br>Recipes	2	2	0.0208	CMI<br>LPN	Tai Yuan (Pomnat, 2013)<br>Tai Yuan (Sumridpiem, 2017)
**Passifloraceae**										
163) *Passiflora foetida* L.	Climber*	Leaves,<br>Inflorescences,<br>Infructescences	Decoction	Oral ingestion	Single used	3	1	0.0104	MSN	Tai Yai (Udompanid, 2012)
**Phyllanthaceae**										
164) *Antidesma acidum* Retz.	Shrub	Roots<br>Roots<br>Roots	Decoction<br>Decoction<br>Decoction	Oral ingestion<br>Oral ingestion<br>Oral ingestion	Recipes<br>Recipes<br>Recipes	3	3	0.0312	MSN<br>MSN<br>MSN	Tai Yuan (Inta et al., 2020)<br>Karen (Inta et al., 2020)<br>Lua (Inta et al., 2020)
165) *Bischofia javanica* Blume	Tree	Non-aerial parts	Decoction	Bath	Recipes	2	2	0.0208	CMI	P. Sumridpiem 2022 15/ Tai Yai (N=2)
166) *Phyllanthus acidus* (L.) Skeels	Tree*	Bark, Roots<br>Stem<br>Infructescences	Decoction<br>Decoction<br>Fresh	Oral ingestion<br>Oral ingestion<br>Oral ingestion	Recipes<br>Single used<br>Single used	4	3	0.0312	CMI<br>CMI<br>MSN	Karen (Winijchaiyanan, 1995)<br>Tai Yuan (Panyadee et al., 2019)<br>Tai Yuan (Inta et al., 2020)
167) *Phyllanthus amarus* Schumach. & Thonn.	Herb	Entire plant<br>Entire plant<br>Entire plant<br>Entire plant<br>Entire plant	Decoction<br>Decoction<br>Decoction<br>Decoction<br>Decoction	Oral ingestion<br>Oral ingestion<br>Oral ingestion<br>Oral ingestion<br>Oral ingestion	Single used<br>Single used<br>Single used<br>Single used<br>Single used	6	6	0.0625	LPN<br>LPN<br>PYO, PRE<br>MSN<br>LPN	Tai Yuan (Inta et al., 2013B)<br>Tai Yuan (Sumridpiem, 2017)<br>Tai Yuan (Inta et al., 2019)<br>Karen (Inta et al., 2020)<br>Tai Yuan (Inta et al., 2020)
168) *Phyllanthus emblica* L.[a]	Tree	Infructescences<br>-<br>Infructescences<br>Galls	Fresh<br>-<br>Fresh<br>Fresh	Oral ingestion<br>-<br>Oral ingestion<br>Oral ingestion	Single used<br>Recipes<br>Single used<br>Single used	4	4	0.0416	LPN<br>LANNA<br>CMI<br>CMI	Tai Yuan (Inta et al., 2013B)<br>Tai Yuan (Manosroi et al., 2013)<br>Tai Yuan (Pomnat, 2013)<br>Lisu (Panyadee et al., 2019)
169) *Phyllanthus urinaria* L.[a]	Herb	Entire plant	Decoction	Oral ingestion	Single used	1	1	0.0104	LPN	Tai Yuan (Sumridpiem., 2017)
**Pinaceae**										
170) *Pinus kesiya* Royle ex Gordon[a]	Tree	Stem (with o il)	Fresh, Decoction	Inhalation, Herbal steam	Single used,<br>Recipes	6	6	0.0625	CMI	P. Sumridpiem 2023 15/TAI YAI (N=1), Karen (N=4), Lisu (N=1)
171) *Pinus latteri* Mason[a]	Tree	Stem (with oil),<br>Resin	Fresh, Decoction	Inhalation,<br>Herbal steam	Single used,<br>Recipes	7	7	0.0729	CMI	Karen (Biodiversity-based economy development office, 2022)<br>P. Sumridpiem 2023 16/ Tai Yai (N=1), Karen (N=4), Lisu (N=1)
**Piperaceae**										
172) *Peperomia pellucida* (L.) Kunth[a]	Herb	Entire plant,<br>Non-aerial parts	Decoction	Oral ingestion	Single used,<br>Food	2	1	0.0104	LPN	Tai Yuan (Sumridpiem, 2017)
173) *Piper interruptum* Opiz[a]	Climber*	Stem	Decoction<br>(with sugar)	Oral ingestion	Single used	1	1	0.0104	TAK	Karen (Kantasrila, 2016)
174) *Piper longum* L.[a]	Herb	-	-	-	Recipes	1	1	0.0104	LANNA	Tai Yuan (Manosroi et al., 2013)
175) *Piper nigrum* L.[a]	Climber*	Infructescences<br>-<br>Infructescences	Fresh<br>-<br>Decoction,<br>Herbal liquor	Oral ingestion<br>-<br>Oral ingestion,<br>Herbal steam, Bath	Single used<br>Recipes<br>Recipes	10	4	0.0416	LPN<br>LANNA<br>CMI	Tai Yuan (Inta et al., 2013B)<br>Tai Yuan (Manosroi et al., 1994)*<br>P. Sumridpiem/ Lisu (N=2)
176) *Piper retrofractum* Vahl[a]	Climber*	Infructescences<br>Infructescences	Decoction<br>Decoction	Oral ingestion<br>Oral ingestion	Single used<br>Recipes	2	2	0.0208	LPN<br>PYO	Tai Yuan (Inta et al., 2013B)<br>Tai Yuan (Inta et al., 2019)
177) *Piper ribesioides* Wall.	Climber	-	-	-	Recipes	1	1	0.0104	LANNA	Tai Yuan (Manosroi et al., 2013)
178) *Piper sarmentosum* Roxb.[a]	Climber	Leaves	Fresh	Oral ingestion	Single used	1	1	0.0104	NAN	Tai Yuan (Songsangchu, 2015)
**Plantaginaceae**										
179) *Plantago major* L.	Herb	Leaves	Fresh	Oral ingestion	Single used	1	1	0.0104	CMI	Hmong (Nguanchoo, 2014)

Scientific Name	Habit	Part	Preparation	Application	Type of medicine	Use reports	FC	RFC	Study area (Province)	Ethnic (References)/Voucher number
180) *Scoparia dulcis* L.	Herb*	Leaves<br>Entire plant	Grinding<br>Dried and decoction	Oral ingestion<br>Oral ingestion	Single used<br>Single used	2	2	0.0208	NAN<br>TAK	Karen (Kantasrila, 2016)<br>Mien (Srithi, 2012)
**Plumbaginaceae**										
181) *Plumbago indica* L.[a]	Shrub	Roots	Decoction	Oral ingestion	Recipes	1	1	0.0104	CMI	P. Sumridpiem 2022 78 / Tai Yai (N=1)
182) *Plumbago zeylanica* L.	Shrub	Shrub	-	-	Recipes	1	1	0.0104	LANNA	Tai Yuan (Manosroi et al., 2013)
**Poaceae**										
183) *Chrysopogon zizanioides* (L.) Roberty [a]	Grass	-	-	-	Recipes	1	1	0.0104	LANNA	Tai Yuan (Manosroi et al., 2013)
184) *Coix lacryma* – jobi L.	Grass*	Inflorescences<br>Roots<br>Roots<br>Roots	Decoction<br>Decoction<br>Decoction<br>Decoction	Oral ingestion<br>Oral ingestion<br>Oral ingestion<br>Oral ingestion	Single used<br>Recipes<br>Recipes<br>Recipes	4	4	0.0416	LPN<br>MSN<br>MSN<br>MSN	Tai Yuan (Sumridpiem, 2017)<br>Tai Yuan (Inta et al., 2020)<br>Karen (Inta et al., 2020)<br>Lawa (Inta et al., 2020)
185) *Coix lacryma*-jobi var. *puellarum* (Balansa) E.G. Camus & A.Camus	Grass*	Entire plant	Decoction	Oral ingestion	Single used	1	1	0.0104	LPN	Tai Yuan (Inta et al., 2013B)
186) *Cymbopogon citratus* (DC.) Stapf [a]	Grass*	Leaves<br>Leaves<br>Entire plant<br>Entire plant<br>Leaves,<br>Entire plant	Decoction<br>Decoction<br>Decoction<br>Decoction<br>Burning,<br>Cooking	Oral ingestion<br>Oral ingestion<br>Oral ingestion<br>Oral ingestion<br>Oral ingestion ,<br>Poultices	Single used<br>Single used<br>Single used<br>Single used<br>Food, Single used	6	6	0.0625	LPN<br>LPN<br>MSN<br>MSN<br>CMI	Tai Yuan (Inta et al., 2013B)<br>Tai Yuan (Sumridpiem (2017)<br>Tai Yuan (Inta et al., 2020)<br>Karen (Inta et al., 2020)<br>P. Sumridpiem/ Hmong (N=2)
187) *Oryza sativa* L.	Grass	Seed<br>-<br>Seed	-<br>-<br>Decoction	-<br>-<br>Herbal Steam	Recipes<br>Recipes<br>Recipes	3	3	0.0312	NAN<br>LANNA<br>CMI	H'Tin (Pantarod, 2002)<br>Tai Yuan (Manosroi et al., 2013)<br>P. Sumridpiem 2022 61/ Tai Yai (N=1)
188) *Saccharum officinarum* L.	Grass	Stem<br>Stem<br>Stem<br>-<br>Stem	Decoction<br>-<br>Decoction<br>-<br>Decoction,<br>Infusion (honey)	Oral ingestion<br>-<br>Oral ingestion<br>-<br>Herbal Steam,<br>Oral ingestion	Single used<br>Recipes<br>Recipes<br>Recipes<br>Recipes	6	6	0.0625	LPN<br>PRE<br>LPN<br>LANNA<br>CMI	Tai Yuan (Inta et al., 2013B)<br>Tai Yuan (Inta et al., 2019)<br>Tai Yuan (Sumridpiem, 2017)<br>Tai Yuan (Manosroi et al., 2013)<br>P. Sumridpiem/ Tai Yai (N=1), /Karen (N=1)
189) *Zea mays* Limm.[a]	Grass*	Inflorescences (Style)	Drying,<br>Decoction,<br>Burning	Oral ingestion,<br>Inhalation,<br>Fumigate	Single used	4	1	0.0104	LANNA	Tai Yuan (Manosroi et al., 2009)
**Polygalaceae**										
190) *Polygala chinensis* L.	Herb	Entire plant	Decoction	Oral ingestion	Single used	1	1	0.0104	CMI	P. Sumridpiem 2022 71/Karen (N=1)
**Polypodiaceae**										
191) *Platycerium wallichii* Hook.	Epiphytic Fern	Leaves<br>Leaves	Decoction<br>Burning,<br>Decoction	Streaming<br>Poultices,<br>Herbal Steam	Single used<br>Recipes, Single used	2	3	0.0312	TAK<br>CMI	Karen (Kantasrila, 2016)<br>P. Sumridpiem /Karen (N=1), Hmong (N=1)
**Ranunculaceae**										
192) *Nigella sativa* L. [a]	Herb*	Seed<br>Seed	Powdering<br>Decoction	Inhalation, Spray<br>Herbal Steam	Recipes<br>Recipes	3	2	0.0208	LANNA<br>CMI	Tai Yuan (Manosroi et al., 1994)*<br>P. Sumridpiem 2022 51/ Tai Yai(N=1)
**Rhamnaceae**										
193) *Colubrina asiatica* (L.) Brongn.	Climber	Leaves, shoots	Pounding	Oral ingestion	Recipes	2	1	0.0104	LPN	Tai Yuan (Sumridpiem, 2017)
194) *Ziziphus jujuba* Mill.	Shrub*	Bark	Grinding	Oral ingestion	Recipes	1	1	0.0104	CMI	P. Sumridpiem 2022 62/ Tai Yai (N=1)

Scientific Name	Habit	Part	Preparation	Application	Type of medicine	Use reports	FC	RFC	Study area (Province)	Ethnic (References)/Voucher number
**Rubiaceae**										
195) *Ixora cibdela* Craib	Shrub	Roots, Leaves	Decoction, Infusion	Oral ingestion, Herbal steam, Bath	Single used, Recipes	9	3	0.0312	CMI	P. Sumridpiem 2022 18/Lisu (N=3)
196) *Morinda angustifolia* Roxb.	Shrub	Roots<br>Roots<br>Roots<br>Leaves	Decoction<br>Decoction<br>Decoction<br>Decoction	Oral ingestion<br>Oral ingestion<br>Oral ingestion<br>Herbal steam	Recipes<br>Single used<br>Single used<br>Recipes	4	4	0.0416	CMI<br>NAN<br>NAN<br>CMI	Karen (Winijchaiyanan, 1995)<br>Khamu (Srithi, 2012)<br>Hmong (Srithi, 2012)<br>P. Sumridpiem 2022 48 / Karen (N=1)
197) *Morinda citrifolia* L.[a]	Tree	Infructescences<br>Infructescences<br>Infructescences<br>Infructescences<br>Infructescences<br>Infructescences	Fresh<br>Cooking<br>Infusion (honey)<br>Infusion (salt)<br>Fresh<br>Decoction	Oral ingestion<br>Oral ingestion<br>Oral ingestion<br>Oral ingestion<br>Oral ingestion<br>Oral ingestion	Single used<br>Food<br>Single used<br>Single used<br>Single used<br>Single used	6	3	0.0312	LPN<br>LPN<br>LPN<br>LPN<br>LPN<br>PYO	Tai Yuan (Sumridpiem, 2017)<br>Tai Yuan (Sumridpiem, 2017)<br>Tai Yuan (Sumridpiem, 2017)<br>Tai Yuan (Sumridpiem, 2017)<br>Tai Yuan (Inta et al., 2013B)<br>Tai Yuan (Inta et al., 2019)
**Rutaceae**										
198) *Aegle marmelos* (L.) Corrêa ex Roxb.[a]	Tree	Infructescences<br>Infructescences<br>Infructescences<br>-	Decoction<br>Decoction<br>Decoction<br>-	Oral ingestion<br>Oral ingestion<br>Oral ingestion<br>-	Single used<br>Single used<br>Single used<br>Recipes	4	4	0.0416	LPN<br>MSN<br>MSN<br>LANNA	Tai Yuan (Inta et al., 2013B)<br>Karen (Inta et al., 2020)<br>Tai Yuan (Inta et al., 2020)<br>Tai Yuan (Manosroi et al., 2013)
199) *Citrus aurantiifolia* (Christm.) Swingle[a]	Shrub*	Infructescences<br>-<br>Infructescences	Crushing (with honey)<br>-	Oral ingestion<br>-<br>Oral ingestion, Inhalation	Single used<br>Recipes<br>Single used	4	4	0.0416	CMI<br>LANNA<br>CMI	Tai Yuan (Inta et al., 2013A)<br>Tai Yuan (Manosroi et al., 2013)<br>P. Sumridpiem /Lisu (N=1), Karen (N=1)
200) *Clausena harmandiana* (Pierre) Guillaumin	Shrub	Leaves	Fresh	Oral ingestion	Single used	1	1	0.0104	MSN	Tai Yuan (Inta et al., 2020)
201) *Harrisonia perforata* (Blanco) Merr.	Shrub	-	-	-	Recipes	1	1	0.0104	LANNA	Tai Yuan (Manosroi et al., 2013)
202) *Melicope pteleifolia* (Champ. ex Benth.) T.G.Hartley	Shrub	Roots<br>Roots<br>Leaves	Decoction<br>Decoction<br>Decoction	Oral ingestion<br>Oral ingestion<br>Herbal steam	Single used<br>Single used<br>Recipes	3	3	0.0312	MSN<br>MSN<br>CMI	Tai Yuan (Inta et al., 2020)<br>Lua (Inta et al., 2020)<br>P. Sumridpiem 2022 47 /Lisu (N=1)
203) *Zanthoxylum asiaticum* (L.) Appelhans, Groppo & J. Wen	Climber	Stem	Infusion, Decoction	Oral ingestion, Herbal steam, Bath	Recipes	8	2	0.0208	CMI	P. Sumridpiem 2022 46 /Lisu (N=2)
204) *Zanthoxylum rhetsa* (Roxb.) DC.	Tree	Infructescences	-	Oral ingestion	Recipes	1	1	0.0104	MSN	Tai Yuan (Inta et al., 2020)
**Salicaceae**										
205) *Flacourtia indica* (Burm.f.) Merr.	Tree	Bark	Grinding	Oral ingestion	Recipes	1	1	0.0104	CMI	P. Sumridpiem 2022 42 / Tai Yai (N=1)
**Salvadoraceae**										
206) *Azima sarmentosa* (Blume) Benth. & Hook.f.	Climber	-	-	-	Recipes	1	1	0.0104	LANNA	Tai Yuan (Manosroi et al., 2013)
**Santalaceae**										
207) *Santalum album* L.	Herb*	Stems	Grinding	Oral ingestion	Recipes	1	1	0.0104	CMI	P. Sumridpiem 2022 83 / Tai Yai (N=1)
**Sapindaceae**										
208) *Cardiospermum halicacabum* L.	Climber	Entire plant	Decoction	Oral ingestion	Single used	2	1	0.0104	PYO, PRE	Tai Yuan (Inta et al., 2019)
**Sapotaceae**										
209) *Mimusops elengi* L.[a]	Tree	-	-	-	Recipes	1	1	0.0104	LANNA	Tai Yuan (Manosroi et al., 2013)

Scientific Name	Habit	Part	Preparation	Application	Type of medicine	Use reports	FC	RFC	Study area (Province)	Ethnic (References)/Voucher number
**Saururaceae**										
210) *Houttuynia cordata* Thunb.	Herb	Leaves<br>Leaves<br>Leaves<br>Leaves<br>Roots, Entire plant	Fresh<br>Fresh<br>Fresh<br>Fresh<br>Infusion (honey), Fresh	Oral ingestion<br>Oral ingestion<br>Oral ingestion<br>Oral ingestion<br>Oral ingestion	Food<br>Food<br>Single used<br>Single used<br>Recipes, Food	6	6	0.0625	NAN<br>LPN<br>MSN<br>MSN<br>CMI	Mien (Srithi, 2012)<br>Tai Yuan (Sumridpiem, 2017)<br>Tai Yuan (Inta et al., 2020)<br>Karen (Inta et al., 2020)<br>P. Sumridpiem 2022 11/ Karen (N=1), Tai Yai (N=1)
**Scrophulariaceae**										
211) *Buddleja asiatica* Lour.	Shrub	Leaves	Decoction	Herbal steam	Recipes	1	1	0.0104	CMI	P. Sumridpiem 2022 26/ Lisu (N=1)
**Smilacaceae**										
212) *Smilax ovalifolia* Roxb. ex D.Don	Climber	Stem	Decoction	Oral ingestion	Single used	1	1	0.0104	LPN	Tai Yuan (Inta et al., 2013B)
213) *Smilax verticalis Gagnep.*	Shrub	Stem	Decoction	Oral ingestion	Single used	1	1	0.0104	LPN	Tai Yuan (Inta et al., 2013B)
**Solanaceae**										
214) *Capsicum frutescens* L.	Shrub*	-	-	-	Recipes	1	1	0.0104	LANNA	Tai Yuan (Manosroi et al., 2013)
215) *Solanum americanum* Mill.	Herb*	Infructescences	Fresh<br>Fresh	Oral ingestion<br>Oral ingestion	Single used<br>Single used	2	2	0.0208	MSN<br>MSN	Lawa (Inta et al., 2020)<br>Karen (Inta et al., 2020)
216) *Solanum spirale* Roxb.	Shrub	Infructescences	Fresh	Oral ingestion	Food	1	1	0.0104	NAN	Mien (Srithi, 2012)
217) *Solanum trilobatum* L.	Climber	-	-	-	Recipes	1	1	0.0104	LANNA	Tai Yuan (Manosroi et al., 2013)
218) *Solanum torvum* Sw.	Shrub*	Infructescences<br><br>Infructescences<br>Infructescences<br>Infructescences	Cooking, Fresh<br>Fresh<br>Fresh<br>Fresh	Oral ingestion<br><br>Oral ingestion<br>Oral ingestion<br>Oral ingestion	Single used, Food<br>Single used<br>Single used<br>Single used	5	4	0.0416	LPN<br><br>MSN<br>MSN<br>MSN	Tai Yuan (Inta et al., 2013B)<br><br>Tai Yuan (Inta et al., 2020)<br>Lawa (Inta et al., 2020)<br>Karen (Inta et al., 2020)
219) *Solanum violaceum* Ortega	Shrub	Infructescences<br>Infructescences	Fresh<br>Fresh	Oral ingestion<br>Oral ingestion	Food<br>Food	2	2	0.0208	MSN<br>MSN	Tai Yuan (Inta et al., 2020)<br>Karen (Inta et al., 2020)
**Urticaceae**										
220) *Elatostema longipes* W.T.Wang	Herb	Leaves	Cooking	Oral ingestion	Food	1	1	0.0104	NAN	Hmong (Srithi, 2012)
221) *Debregeasia longifolia* (Burm.f.) Wedd.	Shrub	Leaves, Shoots, Roots	Burning, Decoction	Compress, Oral ingestion	Single used	6	2	0.0208	CMI	P. Sumridpiem 2022 16 /Hmong (N=2)
**Usneaceae**										
222) *Usnea siamensis* Vain.	Lichen	Entire plant	Decoction	Oral ingestion	Single used	1	1	0.0104	CMI	Tai Yuan (Sookchot, 2003)
**Verbenaceae**										
223) *Tectona grandis* L.f.	Tree	Stem	Decoction	Oral ingestion	Single used	1	1	0.0104	LPN	Tai Yuan (Inta et al., 2013B)
224) *Verbena officinalis* L.	Herb*	Entire plant	Decoction	Herbal steam	Recipes	1	1	0.0104	CMI	P. Sumridpiem 2022 81/ Tai Yai (N=1)
**Viburnaceae**										
225) *Sambucus javanica* Reinw. ex Blume	Shrub	Leaves	Burning	Poultices	Recipes	2	2	0.0208	CMI	P. Sumridpiem 2022 5/ Lisu (N=2)
**Vitaceae**										
226) *Leea indica* (Burm. f.) Merr.	Shrub	Roots	Decoction	Oral ingestion	Single used	1	1	0.0104	NAN	Hmong (Srithi, 2012)
**Zingiberaceae**										
227) *Alpinia galanga* (L.) Willd.[a]	Herb*	Rhizome	-<br>Decoction, Pounding	-<br>Liniment, Oral ingestion	Recipes<br>Recipes	3	3	0.0312	LANNA<br>CMI	Tai Yuan (Manosroi et al., 2013)<br>P. Sumridpiem/ Tai Yai (N=1), Hmong (N=1)
228) *Alpinia officinarum* Hance	Herb*	-	-	-	Recipes	1	1	0.0104	LANNA	Tai Yuan (Manosroi et al., 2013)
229) *Boesenbergia rotunda* (L.) Mansf.	Herb	-	-	-	Recipes	1	1	0.0104	LANNA	Tai Yuan (Manosroi et al., 2013)
230) *Curcuma longa* L.[a]	Herb	Rhizome	Decoction, Pounding	Liniment (temporal area), Oral ingestion	Recipes, Single used	3	3	0.0312	CMI	P. Sumridpiem 2022 35 / Tai Yai (N=1), Hmong (N=2)
231) *Kaempferia galanga* L.	Herb	-	-	-	Recipes	1	1	0.0104	LANNA	Tai Yuan (Manosroi et al., 2013)
232) *Kaempferia parviflora* Wall. Ex *Baker*	Herb	Rhizome	-	-	-	1	1	0.0104	MSN	Lisu (Inta et al., 2023)

Scientific Name	Habit	Part	Preparation	Application	Type of medicine	Use reports	FC	RFC	Study area (Province)	Ethnic (References)/Voucher number
233) *Kaempferia roscoeana* Wall.	Herb	-	-	-	Recipes	1	1	0.0104	LANNA	Tai Yuan (Manosroi et al., 2013)
234) *Rhynchanthus longiflorus* Hook.f.	Herb	Rhizome	Decoction	Oral ingestion	Single used	1	1	0.0104	CRI	Akha (Srisanga et al., 2011)
235) *Zingiber officinale* Roscoe.[a,b]	Herb	Rhizome	Powdering Decoction, Pounding Burning	Inhalation, Spray<br>Liniment (temporal area), Poultices	Recipes<br>Recipes, Single used	5	4	0.0416	LANNA<br>CMI	Tai Yuan (Manosroi et al., 1994*)<br>P. Sumridpiem /Hmong (N=3)
236) *Zingiber ottensii* Valeton	Herb	Rhizome	Pounding (with water), Burning	Oral ingestion, Poultices, Compress	Recipes	3	3	0.0312	CMI	P. Sumridpiem 2023 8 / Karen (N=3)
237) *Zingiber purpureum* Roscoe.[a]	Herb	Rhizome	-<br>Burning, Decoction, Soaking, Pounding	-<br>Bath, Compress, Oral ingestion, Poultices, Herbal steam	Recipes<br>Recipes, Single used	21	13	0.1354	LANNA<br>CMI	Tai Yuan (Manosroi et al., 2013)<br>P. Sumridpiem 2023 22/ Karen (N=3), / Tai Yai (N=8), Hmong (N=1)

Scientific Name = ([a]) Listing of a plant that has been previously reported to treat hypertension in any traditional medicine, ([b]) Listing of a plant that has been previously reported for anti-hypertensive properties in any studies, and (**) Listing of references with scientific names from original data sources, such as *Seseli* cf. *mairei* H.Wolff.
Habit = (*) Listing of exotic plant.
Study area = "LANNA" refers to study area from literature review covering Chiang Mai, Chiang Rai, Lampang, Lamphun Phrae Nan, Phayao, or Mae Hong Son.
References = (*) Listing of references with scientific names from tentative identification of the transliterations of herbal medicine recipes that appeared in palm leaf scripture by Lanna folk healers.
Ui = Use reports.
Ethnic (References)/ Voucher number = "N" refers to number of healers from field survey.

Table S2. Details of the 41 references used to analyze northern Thai hypertension plant data.

No.	References	Document Type	Province	District	Ethnicity	Plant Category	Number of species
1	Archam (2011)	Bachelor research	Chiang Mai	Wianghaeng	Shan	Medicine	1
2	Biodiversity-based economy development office (2023)	Book	Chiang Mai, Chiang Rai	Mae Chaem, Mae Fah Luang	Hmong, Karen, Lua (Lawa), Lisu, Lahu, Akha, Mien	Medicine	4
3	Brun and Schmacher (1994)	Book	Chiang Mai, Lamphun	Chiang Dao, Mueang Chiang Mai, Mueang Lamphun, Mae Chaem, Mae Fah Luang	Tai Yuan	Medicine	4
4	Ethnobotany and Northern Thai Flora Laboratory Unit (2015)	Book	Chiang Mai	Mae On	Tai Yuan	All	1
5	Highland Research and Development Institute (2018)	Book	Chiang Mai	Chom Thong	Hmong	Medicine	1
6	Inta et al. (2013A)	Journal article	Chiang Mai	Chiang Dao	Tai Yuan	Medicine	2
7	Inta et al. (2013B)	Journal article	Lamphun	Mueang Lamphun, Mae Tha	Tai Yuan	Medicine	19
8	Inta et al. (2014)	Book	Nan	Phu Phiang	Tai Yuan	All	2
9	Inta et al. (2018A)	Book	Mae Hong Son	Mae Sariang	Karen	Medicine	1
10	Inta et al. (2018B)	Book	Mae Hong Son	Sop Moei	Karen	Medicine	1
11	Inta et al. (2018C)	Book	Mae Hong Son	Khun Yuam	Karen	Medicine	1
12	Inta et al. (2018D)	Book	Mae Hong Son	Khun Yuam	Shan	Medicine	1
13	Inta et al. (2019)	Book	Phayao, Phrae	Mueang Phayao, Chiang Kham, Chun, Chiang Muan, Pong, Phu Sang, Dok Khamtai, Mae Chai, Mueang Phrae, Wang Chin, Song	Tai Yuan	Medicine	13

Table S2. Details of the 41 references used to analyze northern Thai hypertension plant data (cont.).

No.	References	Document Type	Province	District	Ethnicity	Plant Category	Number of species
14	Inta et al. (2020)	Book	Mae Hong Son, Lamphun	Mueang Mae Hong Son, Khun Yuam, Pang Ma Pha, Pai, Mae La Noi, Mae Sariang, Sop Moei, Mueang Lamphun, MaeTha, Ban Hong, Li Thung, Hua Chang, Pa Sang, Ban Thi, Wiang Nong Long	Tai Yuan, Karen, Lawa, Shan	Medicine	24
15	Inta et al. (2023)	Journal article	Mae Hong Son	Pang Mapha	Lisu	Zingiberaceae family	1
16	Kaewsangsai (2017)	Thesis (M.Sc.)	Chiang Mai	Omkoi	Karen	All	1
17	Kantasrila (2016)	Thesis (M.Sc.)	Tak	Tha Song Yang	Karen	All	7
18	Manosroi and Manosroi (1994)	Book	Lanna (Chiang Mai, Chiang Rai, Lampang, Lamphun Phrae Nan, Phayao, Mae Hong Son)	Not reported	Tai Yuan	Medicine	9
19	Manosroi et al. (2009)	Book	Lanna (Chiang Mai, Chiang Rai, Lampang, Lamphun Phrae Nan, Phayao, Mae Hong Son)	Not reported	Tai Yuan	Medicine	2
20	Manosroi et al. (2013)	Journal article	Lanna (Chiang Mai, Chiang Rai, Lampang, Lamphun Phrae Nan, Phayao, Mae Hong Son)	Not reported	Tai Yuan	Medicine	47
21	Muangyen (2013)	Thesis (M.Sc.)	Chiang Mai	Samoeng	Tai Lue	All	4
22	Nguanchoo (2014)	Thesis (M.Sc.)	Chiang Mai	Mae Rim	Hmong	All	5
23	Panta (2015)	Bachelor research	Chiang Mai	Phrao	Lisu	All	1
24	Pantarod (2002)	Thesis (M.Sc.)	Nan	Bo Kluea	H'Tin	Medicine	1
25	Panyadee et al (2019)	Journal article	Chiang Mai	Omkoi , Phrao, Mae Chaem	Tai Yuan, Karen, Lisu, Lahu	Medicine	3

Table S2. Details of the 41 references used to analyze northern Thai hypertension plant data (cont.).

No.	References	Document Type	Province	District	Ethnicity	Plant Category	Number of species
26	Panyaphu (2012)	Thesis (Ph.D.)	Nan	Tha Wang Pha	Mien	Medicine	1
27	Phongloy (2015)	Thesis (M.Sc.)	Mae Hong Son, Chiang Mai	Khun Yuam, Wianghaeng	Shan	All	4
28	Pomnat (2013)	Bachelor research	Chiang Mai	Hang Dong	Tai Yuan	All	3
29	Siewphukhieo (1987)	Thesis (M.Sc.)	Chiang Mai	Muang Chiang Mai	Hmong	Non-Food Wild Plants	1
30	Songsangchun (2015)	Thesis (M.Sc.)	Nan	Bo Klua	Tai Yuan	All	2
31	Sookchot (2003)	Bachelor research	Chiang Mai	Chiang Dao	Tai Yuan	Medicine	1
32	Srisanga et al. (2011)	Journal article	Chiang Rai	Mae Suai	Akha	All	3
33	Srithi (2012)	Thesis (Ph.D.)	Nan	Mueang Nan, Pua, Thung Chang, Song Khwae	Mien, Khamu, Lawa, Hmong	All	17
34	Sukkho (2008)	Thesis (M.Sc.)	Chiang Mai	Mae Chaem	Karen	Medicine	1
35	Sumridpiem (2017)	Thesis (M.Sc.)	Lamphun	Mueang Lamphun, Pa Sang	Tai Yuan, Tai Yong	All	37
36	Sutjaritjai (2019)	Thesis (Ph.D.)	Chiang Mai	Samoeng, Omkoi, Chom Thong, Mae Chaem	Karen (Pao), Karen (Sakao)	All	7
37	Tangjitman (2014)	Thesis (M.Sc.)	Chiang Mai	Samoeng	Karen	Medicine	1
38	Tovaranonte (1998)	Thesis (M.Sc.)	Nan	Tha Wang Pha, Pua	Lawa, Hmong, Mien	All	2
39	Tovaranonte (2002)	Research reports	Chiang Rai	Mueang Chiang Rai	Tai Yuan	All	3
40	Udompanid (2012)	Bachelor research	Mae Hong Son	Mueang Mae Hong Son	Shan	All	1
41	Winijchaiyanan (1995)	Thesis (M.Sc.)	Chiang Mai	Mae Chaem, Mae Tang	Karen	All	6

References

1. Archam, N. 2011. Traditional Medicinal Plant of Tai Yai in Laktaeng Village, Wiang Haeng District, Chiang Mai Province., B.Sc. Special project (Biology). Chiang Mai University.
2. Biodiversity-based economy development office, 2023. Folk wisdom in Healthcare of ethnic groups in the northern region, Thailand: Wanida printing [in Thai].
3. Brun, V., Schmacher, T., 1994. Traditional Herbal Medicine in Northern Thailand. White Lotus Co., Ltd. Bangkok, Thailand.
4. Ethnobotany and Northern Thai Flora Laboratory Unit. 2015. Development plan for conservation and sustainable utilization of biological resources and local wisdom of Mae Tha community, Mae Tha Subdistrict, Chiang Mai Province. Ethnobotany and Northern Thai Flora Laboratory, Chiang Mai University. Biodiversity-based economy development office, Thailand: Wanida printing [in Thai].
5. Highland Research and Development Institute (Public Organization). 2018. The Guide of local plants utilization of Hmong in Ban PaKlwy Phathna, Mae Soi Subdistrict, Chom Thong District, Chiang Mai Province: Wanida printing [in Thai].
6. Inta, A., Sirisa-ard, P., Pongamornkul, W., 2013A. Medicinal plants in ban hua thung community forest, Chiang Dao wildlife sanctuary, Chang Dao district, Chiang Mai province. Thail. J. Bot. 4, 213–232.
7. Inta, A., Trisonthi, P., Trisonthi, C., 2013B. Analysis of traditional knowledge in medicinal plants used by Yuan in Thailand. J. Ethnopharmacol. 149, 344–351.
8. Inta. A., Srisanga. P., Panyadee, P., Pongamornkul. W. 2018A. Biodiversity of medicinal plants in Mae Han community forest, Sop Moei Subdistrict, Sop Moei District, Mae Hong Son Province: Wanida printing [in Thai].
9. Inta. A., Srisanga. P., Panyadee, P., Pongamornkul. W. 2018B. Biodiversity of medicinal plants in Mae Khatuan community forest, Sop Moei Subdistrict, Sop Moei District, Mae Hong Son Province: Wanida printing [in Thai].
10. Inta. A., Srisanga. P., Panyadee, P., Pongamornkul. W. 2018C. Biodiversity of medicinal plants in Mae Ki community forest, Mae Ki Subdistrict, Khun Yuam District, Mae Hong Son Province: Wanida printing [in Thai].
11. Inta. A., Srisanga. P., Panyadee, P., Pongamornkul. W. 2018D. Biodiversity of medicinal plants in Tau Pae community forest, Mae Nga Subdistrict, Khun Yuam District, Mae Hong Son Province: Wanida printing [in Thai].
12. Inta. A., Srisanga. P., Pongamornkul. W., Khongkeaw. B., Muangmun. N., Muangmun. W., 2019. The biological resources inventory of medicinal plants and folk wisdom in Phayao and Phrae Province. Chiang Mai University. Biodiversity-based economy development office, Thailand: Wanida printing [in Thai].
13. Inta. A., Srisanga. P., Pongamornkul. W., Khongkeaw. B., Muangmun. N., Muangmun. W., 2020. The biological resources inventory of medicinal plants and folk wisdom in Mae Hong Son and Lamphune Province. Chiang Mai University. Biodiversity-based economy development office, Thailand: Wanida printing [in Thai].
14. Inta. A., Trisonthi, P., Srithi. K., Pongamornkul. W. 2014. Biodiversity and folk wisdom of development plan for conservation and sustainable utilization of biological resources in Nam Kian community, Nam Kian Subdistrict, Nan Province. Chiangmai documentary design company limited printing [in Thai].
15. Inta A. , Trisonthi C. , Pongamornkul W. , Panyadee P. 2023. Ethnobotany of Zingiberaceae in Mae Hong Son, Northern Thailand. Biodiversitas. 24:2114-2124.
16. Kaewsangsai, S. 2017. Ethnobotany of Karen in Khun Tuen Noi Village, Mae Tuen Sub-district, Omkoi District, Chiang Mai Province., M.Sc. Thesis (Biology). Chiang Mai University.
17. Kantasrila, R. 2016. Ehtnobotany of Karen at Ban Wa Do Kro, Mae Song Sub-district, Tha Song Yang District, Tak Province. M.Sc. Thesis (Biology). Chiang Mai University.
18. Manosroi, J. and Manosroi, A. 1994. Lanna Pharmacy: Lanna Medicinal Plants Recipes. Bangkok. The War Veterans Organization of Thailand Under Royal Patronage Printing House [in Thai].

19. Manosroi, J., Manosroi, A. and Rungruangsri, U., 2009. Lanna Medicinal Plant Dictionary. Pharmaceutical-Cosmetic Raw Materials and Natural Products Research and Development Center (PCRNC), Institute for Science and Technology Research and Development (IST), Chiang Mai University, Thailand.
20. Manosroi, A., Lohcharoenkal, L., Khonsung, P., Manosroi, W. and Manosroi, J. 2013. Potent antihypertensive activity of Thai-Lanna medicinal plants and recipes from MANOSROI III database. Pharmaceutical Biology. 51(11): 1426 –1434.
21. Muangyen, N., 2013. Ethnobotany of Tai Lue and Tai Yuan in Samoeng District, Chiang Mai Province. Master thesis (Biology). Chiang Mai University.
22. Nguanchoo, V. 2014. Ethnobotany of Hmong in Mae Rim District, Chiang Mai Province, Thailand. M.Sc. Thesis (plant sciene). Mahidol University.
23. Panta, K. 2015. Ethnobotany of Lisu in Khun Chae Village, Phrao District, Chiang Mai B.Sc. Special project (Biology). Chiang Mai University.
24. Pantarod, B., 2002. A survey and collection of medicinal plants at Na Kwang village, Bo Kleau district, Nan province. M.Sc. Thesis (Biology). Chiang Mai University.
25. Panyadee, P., Balslev, H. Wangpakapattanawong, P., & Inta, A. 2019. Medicinal plants in homegardens of four ethnic groups in Thailand. Journal of Ethnopharmacology. 239, 1-14.
26. Panyaphu, K., 2012. Conservation and sustainable use of ethnomedicinal plants by Mien people in Nan Province. PhD thesis, Biology. Chiang Mai University Library, Chiang Mai.
27. Phongloy, T. 2015. Biodiversity and Utilization of plants from protected and utilized forests by Tai Yai communities in Chiang Mai and Mae Hong Son Provinces. Ph.D. Thesis (Biology). Chiang Mai University.
28. Pomnat, S. 2013. Ethnobotany of Ban Pong Nuea Village, Hang Dong District, Chiang Mai Province. B.Sc. Special project (Biology). Chiang Mai University.
29. Siewphukhieo, S. 1987 Non-Food Wild Plants Used by Hill Tribes and Local People in Some Area of Changwat Chiang Mai. M.Sc. Thesis (Biology). Chiang Mai University.
30. Songsangchun, A. 2015. Plants usages of Khon Muang and Lawa in Phu Fah subdistrict, Bo Klua district, Nan province M.Sc. Thesis (Biology). Chiang Mai University.
31. Sookchot, T., 2003. Study of Medicinal Plants in Sold at Ban Thum, Chiang Dao District, Chiang Mai Province B.Sc. Special project (Biology). Chiang Mai University.
32. Srisanga, P., Wongpakam, S., Kamkuan, W., Pekthong, T., Tovaranonte, J., Yaso, T., Nontachaiyapoom, S., 2011. Ethnobotany of Akha in Huay Yuak Pa So village, Mae Fah Luang district and Ban Mai Patthana village, Mae Suai district, Chiang Rai province. Thai Journal of Botany 3(1), 12.
33. Srithi, K., 2012. Comparative ethnobotany in Nan province, Thailand., Ph.D. Thesis (Biology). Chiang Mai University.
34. Sumridpiem, P. 2018. Utilization Analysis of Medicinal Plants Between Tai Yong and Tai Yuan in Lamphun Province. , M.Sc. Thesis (Biology). Chiang Mai University.
35. Sukkho, T., 2008. A survey of medicinal plants used by Karen people at Ban Chan and Chaem Luang Subdidtricts, Mae Chaem district, Chiang Mai province., M. Sc. (Biology). Chiang Mai University.
36. Sutjaritjai N., 2019. Traditional Uses of Leguminosae among the Karen in Thailand. Ph.D. Thesis (Biology). Chiang Mai University.
37. Tangjitman, K. 2014. Vulnerability predigtion of medicinal plants used by Karen people n Chiang Mai Province to climatic change using Species Distribution Model (SDM). Ph.D. Thesis (Biology). Chiang Mai University.
38. Tovaranonte, J., 1998. Ethnobotanical study of the Tai Lue, Hmong and Yao in Some areas of Nan Province. M.Sc. Thesis (Biology). Chiang Mai University.
39. Tovaranonte J. Ethnobotany in Surrounding Area of Mae Fah Luang University. Mae Fah Luang University, Chiang Rai; 2002.

40. Udompanid, K. 2012. Ethnobotany of Tai Yai in Naisoi Village, Mueng District, Mae Hong Son Province. B.Sc. Special project (Biology). Chiang Mai University.
41. Winijchaiyanan, P., 1995. Ethnobotany of Karen in Chiang Mai. M.Sc. Thesis (Biology). Chiang Mai University.
